# The molecular spectrum of amyloid‐beta (Aβ) in neurodegenerative diseases beyond Alzheimer's disease

**DOI:** 10.1111/bpa.13210

**Published:** 2023-08-31

**Authors:** Shojiro Ichimata, Koji Yoshida, Jun Li, Ekaterina Rogaeva, Anthony E. Lang, Gabor G. Kovacs

**Affiliations:** ^1^ Tanz Centre for Research in Neurodegenerative Disease University of Toronto Toronto Ontario Canada; ^2^ Department of Laboratory Medicine and Pathobiology University of Toronto Toronto Ontario Canada; ^3^ Department of Legal Medicine, Faculty of Medicine University of Toyama Toyama Japan; ^4^ Edmond J Safra Program in Parkinson's Disease and Rossy Program in Progressive Supranuclear Palsy Toronto Western Hospital Toronto Ontario Canada; ^5^ Laboratory Medicine Program and Krembil Brain Institute University Health Network Toronto Ontario Canada

**Keywords:** amyloid‐beta, *apolipoprotein E* ε4, Lewy body disease, progressive supranuclear palsy, sex difference, striatum

## Abstract

This study investigated the molecular spectrum of amyloid‐beta (Aβ) in neurodegenerative diseases beyond Alzheimer's disease (AD). We analyzed Aβ deposition in the temporal cortex and striatum in 116 autopsies, including Lewy body disease (LBD; *N* = 51), multiple system atrophy (MSA; *N* = 10), frontotemporal lobar degeneration‐TDP‐43 (FTLD‐TDP; *N* = 16), and progressive supranuclear palsy (PSP; *N* = 39). The LBD group exhibited the most Aβ deposition in the temporal cortex and striatum (90/76%, respectively), followed by PSP (69/28%), FTLD‐TDP (50/25%), and the MSA group (50/10%). We conducted immunohistochemical analysis using antibodies targeting eight Aβ epitopes in the LBD and PSP groups. Immunohistochemical findings were evaluated semi‐quantitatively and quantitatively using digital pathology. Females with LBD exhibited significantly more severe Aβ deposition, particularly Aβ_42_ and Aβ_43_, along with significantly more severe tau pathology. Furthermore, a quantitative analysis of all Aβ peptides in the LBD group revealed an association with the *APOE‐*ε4 genotypes. No significant differences were observed between males and females in the PSP group. Finally, we compared striatal Aβ deposition in cases with LBD (*N* = 15), AD without α‐synuclein pathology (*N* = 6), and PSP (*N* = 5). There were no differences in the pan‐Aβ antibody (6F/3D)‐immunolabeled deposition burden among the three groups, but the deposition burden of peptides with high aggregation capacity, especially Aβ_43_, was significantly higher in the AD and LBD groups than in the PSP group. Furthermore, considerable heterogeneity was observed in the composition of Aβ peptides on a case‐by‐case basis in the AD and LBD groups, whereas it was relatively uniform in the PSP group. Cluster analysis further supported these findings. Our data suggest that the type of concomitant proteinopathies influences the spectrum of Aβ deposition, impacted also by sex and *APOE* genotypes.

## Graphical abstract

- This study aimed to investigate the molecular spectrum of amyloid‐beta (Aβ) in neurodegenerative diseases beyond Alzheimer's disease (AD).
- The Aβ deposition load in the temporal lobe and striatum is the highest in Lewy body disease (LBD), followed by progressive supranuclear palsy (PSP), frontotemporal lobar degeneration‐TDP, and multiple system atrophy.
- The striatal Aβ peptide spectrum differs between AD, LBD, and PSP.

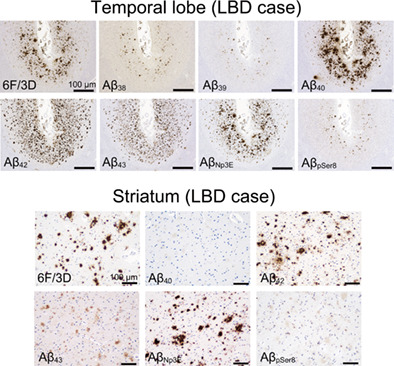

## 1 INTRODUCTION

It is increasingly recognized that combinations of one or more proteinopathies (mixed pathology) frequently occur in individuals with neurodegenerative diseases (NDDs), with the major proteins being amyloid‐beta (Aβ), tau, α‐synuclein, and TAR DNA‐binding protein 43 (TDP‐43) [1, 2, 3, 4, 5, 6]. Aβ deposition is not only the neuropathological hallmark of Alzheimer's disease (AD), but also the principal co‐pathology in several non‐AD NDDs [1, 2, 3, 4, 5]. However, the rate of Aβ deposition varies among these diseases [1]. It is becoming clear that Aβ deposits in AD brains consist of a heterogeneous mixture of Aβ peptides [7, 8]. Therefore, we hypothesized that the molecular signatures of Aβ deposits differ for each proteinopathy.

While age is the most important risk factors for NDDs, sex and certain genotypes, especially *apolipoprotein E* (*APOE*) ε4, are considered significant risk factors for developing late‐onset AD [9, 10, 11, 12]. We recently reported that females with Lewy body disease (LBD) have significantly more severe Aβ deposition, labeled by a pan‐Aβ antibody (clone 6F/3D; epitope lies within 10–15 amino acids) than males [13]. Several studies have also reported sex‐based differences in individuals with LBD, including the concentration of Aβ in cerebrospinal fluid and the severity of AD pathology [14, 15, 16, 17, 18, 19, 20]. Furthermore, *APOE* ε4 status has been reported as a risk factor for the presence of co‐pathologies independent of NDD [3]. Thus, these factors are also speculated to influence the spectrum of Aβ pathology in non‐AD NDDs.

Striatal Aβ deposition is an important pathological finding as it is closely associated with the development of dementia in individuals with LBD [21, 22, 23]. Kalaitzakis et al. reported that the striatal Aβ deposition burden was higher both in Parkinson's disease with dementia (PDD) and dementia with Lewy bodies (DLB) compared to that in PD, multiple system atrophy (MSA), and progressive supranuclear palsy (PSP) [23]. Furthermore, they also reported that striatal cored plaques were only observed in patients with DLB [23]. As the composition of Aβ peptides varies depending on the morphology of the senile plaques [24, 25], it is possible that the composition of striatal Aβ peptides differs in each NDD.

Amyloid‐centric therapeutic strategies have been rapidly evolving [26, 27, 28, 29]. Consequently, it is crucial to comprehend the molecular characteristics of pathological proteins deposited in each non‐AD NDD to enhance therapeutic efficacy. However, there is a lack of knowledge regarding the spectrum of Aβ deposition and its association with some factors such as sex and *APOE* genotype, particularly in non‐AD NDDs. To elucidate this, we conducted a comprehensive immunohistochemical study of non‐AD NDDs, including LBD, MSA, frontotemporal lobar degeneration‐TDP43 (FTLD‐TDP), and PSP. Initially, we performed a semi‐quantitative assessment of Aβ burden in the neocortex and striatum using anti‐pan‐Aβ antibodies (6F/3D) to explore potential differences in Aβ deposition patterns among these diseases. We also assessed the effects of age, sex, and *APOE* genotypes on Aβ deposition in those diseases. Subsequently, we evaluated the spectrum of deposited Aβ peptides using antibodies targeting eight different epitopes of Aβ focusing on LBD and PSP, as these two groups had the most cases in our study. Lastly, we investigated whether the spectrum of deposited Aβ peptides in the striatum differs between LBD, PSP, and AD showing similar levels of 6F/3D‐recognized amyloid deposition by semiquantitative analysis or Thal phase classification [30].

## 2 MATERIALS AND METHODS

### 2.1 Subjects

We reviewed the archives of neuropathological autopsies from the University Health Network Neurodegenerative Brain Collection (UHN‐NBC) and selected 120 consecutive brain donation cases, where, based on clinical and pathological interpretation LBD, MSA, FTLD‐TDP, and PSP were diagnosed. Patients with a clear history of repetitive head injury were excluded from this study Finally, we included 116 cases, including 51 cases of LBD, 10 cases of MSA, 16 cases of FTLD‐TDP, and 39 cases of PSP (Table 1). Regarding the LBD group, we assessed the severity of Lewy‐related pathology using α‐synuclein immunohistochemistry (antibody 5G4) based on the Lewy pathology consensus criteria (LPC) [31, 32]. We included only cases with neocortical and limbic Lewy‐related pathology, as previously described [14]. For comparison, we included 14 AD cases without LBD‐related pathology. All brains were obtained at autopsy through appropriate consenting procedures with Local Ethical Committee approval. This study was approved by the UHN Research Ethics Board (No. 20‐5258) and University of Toronto (No. 39459) and was performed per the ethical standards established in the 1964 Declaration of Helsinki, updated in 2008.

**TABLE 1:** Summary of the cases for the temporal lobe evaluation.

	LBD	MSA	FTLD‐TDP	PSP
No. of cases	51	10	16	39
Sex (F/M/U)	23/28	6/4	6/10/0	16/21/2
Mean age				
All (range)	76.6 ± 8.4 (56–95)	66.8 ± 4.9** (61–76)	69.4 ± 13.4 (46–89)	74.3 ± 6.5^a^ (63–93)
Females (range)	78.8 ± 10.0 (59–95)	68.2 ± 4.8 (61–76)	78.0 ± 5.7 (68–85)	74.3 ± 7.6 (63–93)
Males (range)	74.8 ± 6.3 (56–85)	64.8 ± 4.3 (61–72)	64.3 ± 14.1 (46–89)	74.4 ± 5.4^a^ (63–85)
*APOE* and *MAPT* allele frequency				
No. of available cases (%)	46 (90)	9 (90)	6 (38)	25 (64)
ε2, *n* (%)	1 (1)	1 (6)	4 (33)	6 (12)
ε3, *n* (%)	51 (55)	13 (72)	7 (58)	34 (68)
ε4, *n* (%)	40 (43)	4 (22)	1 (8)*	10 (20)**
H1, *n* (%)	69 (75)	15 (83)	11 (92)	49 (98)**
H2, *n* (%)	23 (25)	3 (17)	1 (8)	1 (2)
NIA‐AA ADNC level (not/low/intermediate/high)				
All (%)	4/13/21/13 (8/25/41/25)	5/5/0/0 (50/50/0/0)	8/6/1/1 (50/38/6/6)	12/20/6/1 (31/51/15/3)
Females (%)	2/3/8/10* (9/13/35/43)	2/4/0/0 (33/67/0/0)	2/3/1/0 (33/50/17/0)	3/9/3/1 (19/56/19/6)
Males (%)	2/10/13/3 (7/36/46/11)	3/1/0/0 (75/25/0/0)	6/3/0/1 (60/30/0/10)	9/9/3/0 (43/43/14/0)
Aβ deposition				
NC‐positive (%)	47 (92)	5 (50)**	8 (50)**	27 (69)*
Str‐positive (%)	39 (76)	1 (10)**	4 (25)**	11 (28)**
Negative (%)	4 (8)	5 (50)	8 (50)	12 (31)
No. of cases with Aβ ≥ A2 and G2 in the temporal lobe				
All (%)	36 (71)	1 (10)	4 (25)	10 (26)
Females (%)	19 (83)	1 (17)	3 (50)	6 (38)
Males (%)	17 (61)	0	1 (10)	4 (19)

Abbreviations: Aβ, amyloid‐beta; ADNC, Alzheimer's disease neuropathologic change; FTLD‐TDP, frontotemporal lobar degeneration‐tans‐activation response DNA‐binding protein 43; LBD, Lewy body disease; MAPT, microtube binding protein tau; MSA, multiple system atrophy; NC, neocortex; NIA‐AA, the National Institute on Aging‐Alzheimer's Association; PSP, progressive supranuclear palsy; Str, striatum; U, unknown.

^a^ Age at death was not available in three cases.

* *p* < 0.05;

** *p* < 0.01.

### 2.2 Tissue sampling and pathological assessment

Formalin‐fixed paraffin‐embedded tissue sections from the temporal lobe (TL) and anterior part of the striatum (including the caudate nucleus [CN], putamen, and nucleus accumbens) were investigated. Immunohistochemistry was performed using 8 different antibodies against different Aβ epitopes, including pan‐Aβ (6F/3D), Aβ_38_, Aβ_39_, Aβ_40_, Aβ_42_, Aβ_43_, pyroglutamate Aβ at 3rd glutamic acid (Aβ_Np3E_), phosphorylated‐(p‐)Aβ at 8th serine (Aβ_pSer8_), and anti‐p‐tau antibodies (Ser202/Thr205, clone AT8). The antibodies and immunostaining methods used in this study are presented in Table 2. Immunostaining was performed using the Dako Autostainer Link 48 and EnVision FLEX+ Visualization System, according to the manufacturer's instructions. Subsequently, all sections were counterstained with hematoxylin.

**TABLE 2:** Summary of antibodies used in this study.

Antibody	Source	Clone	Dilution	First antigen retrieval	Second antigen retrieval
Aβ_aa8–17_	Dako	6F/3D	1:50	80% FA 1 h	None
Aβ_38_	Synaptic Systems	Polyclonal	1:1000	Heat^a^	88% FA 3 min
Aβ_39_	Cell Signaling	D5Y9L	1:500	Heat^a^	88% FA 3 min
Aβ_40_	BioLegend	QA18A67	1:3000	70% FA 10 min	None
Aβ_42_	BioLegend	1‐11‐13	1:500	70% FA 10 min	None
Aβ_43_	IBL	Polyclonal	1:100	88% FA 5 min	None
Aβ_Np3E_	BioLegend	337.48	1:800	Heat^a^	88% FA 3 min
Aβ_pSer8_	Sigma‐Aldrich	1E4E11	1:200	Heat^a^	88% FA 3 min
p‐tau (Ser202, Thr205)	Thermo Fischer	AT8	1:1000	Heat^a^	None

Abbreviations: aa, amino acid; FA, formic acid; p‐tau, phosphorylated‐tau.

^a^ Performed using Dako PT Link with low pH solution.

### 2.3 Neuropathological case selection

**FIGURE 1:**
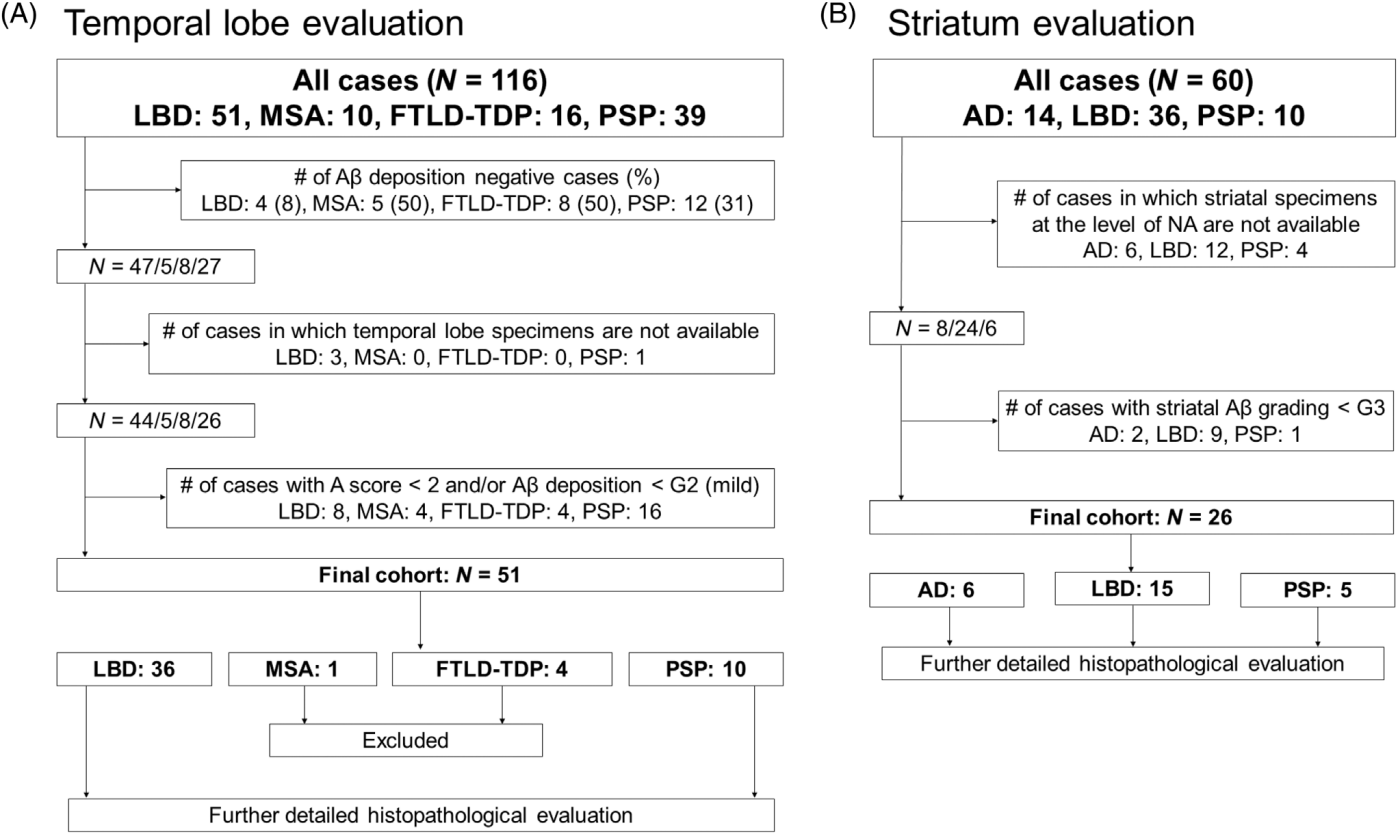
Flow chart of patient and sample selection. Aβ, amyloid‐beta; AD, Alzheimer's disease; FTLD, frontotemporal lobar degeneration; LBD, Lewy body disease; MSA, multiple system atrophy; PSP, progressive supranuclear palsy; TDP, TAR DNA‐binding protein 43.

Following Aβ and tau immunostaining, we classified all cases according to the National Institute on Aging‐Alzheimer's Association (NIA‐AA) guidelines, dividing the level of AD neuropathologic change (ADNC) into four categories (not, low, intermediate, and high) [33]. Additionally, we evaluated the type of cerebral amyloid angiopathy (CAA) according to the Thal classification [34]. For detailed pathological analysis in the TL, we selected cases with an A score of 2 or higher [30, 33] and with mild (G2) or higher Aβ deposition on the 6F/3D‐immunostained specimen (see below). For Aβ analysis in the striatum, we selected cases with moderate (G3) or higher striatal Aβ deposition on the 6F/3D‐immunostained specimen (see below). Figure 1 shows the flow chart of case selection in the TL (Figure 1A) and striatum (Figure 1B).

### 2.4 Semiquantitative grading system for Aβ and tau pathology

**FIGURE 2:**
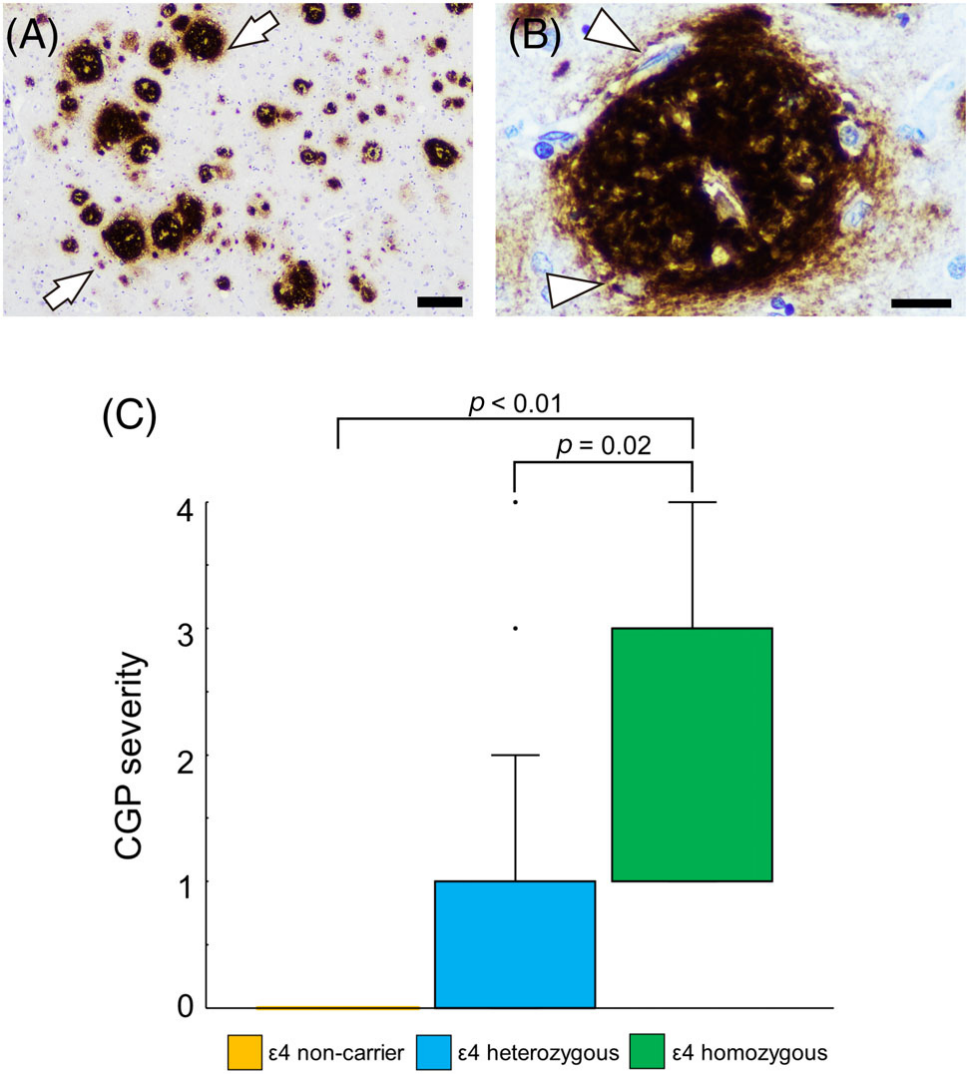
Representative microphotographs of the coarse‐grained plaques (CGPs) and the relationship between the CGP pathological severity and the genotype of *APOE* in individuals with Lewy body disease. (A, B) Immunohistochemistry for 6F/3D. (A) The CGPs are observed as clusters (arrows). (B) The plaque shows multiple cores, Aβ‐devoid pores, a vague rim and tubular‐like and trabecular structures (arrowheads). (C) No CGP formation is observed in non‐APOE ε4 carriers. Statistical analysis was performed using Kruskal–Wallis test with post hoc Bonferroni correction. Scale bar = 200 (A), 20 μm (B).

In addition to the staging systems described above, we semiquantitatively graded the severity of immunohistochemical findings of each Aβ peptide and tau (AT8). The severity of Aβ pathology within brain parenchyma of the TL and striatum (as amyloid plaques) and cerebral vessels (as CAA) was evaluated using a five‐point scoring system [35, 36] as follows: Plaque Grade 0, no Aβ plaques in parenchyma/layer; Grade 1, a few Aβ plaques in parenchyma/layer occupying each low power (×10 microscope objective) field; Grade 2, a moderate number of Aβ plaques in parenchyma/layer occupying each low power field; Grade 3, many dispersed Aβ plaques in parenchyma/layer occupying each low power field; Grade 4, abundant densely packed Aβ plaques in parenchyma/layer occupying each low power field. CAA Grade 0, no CAA in blood vessel walls in leptomeninges or brain parenchyma; Grade 1, occasional blood vessels with CAA in leptomeninges and/or within brain parenchyma, usually not occupying the full thickness of the wall; Grade 2, a moderate number of blood vessels with CAA in leptomeninges or brain parenchyma in leptomeninges or within brain parenchyma, some occupying the full thickness of the wall; Grade 3, many blood vessels with CAA in leptomeninges or brain parenchyma, most occupying the full thickness of the wall; Grade 4, most or all blood vessels with severe CAA in leptomeninges or within brain parenchyma, occupying the full thickness of the wall. The staging system we used for CAA [36] follows the concept of the grading proposed by Love et al. [37] but includes five grades (0–4) instead of four (0–3) and better comparable to the grading we used to evaluate parenchymal deposits. Representative images are shown in Figure S1. Additionally, we evaluated the severity of coarse‐grained plaque (CGP) pathology [24] using the same grading system (representative microphotographs of CGPs are shown in Figure 2A,B).

The severity of overall tau (AT8) pathology, including pre‐tangles, NFTs, and neuropil threads, was evaluated using a five‐point scoring system [35, 36] as follows: Grade 0, no tau pathology present; Grade 1, very few and scattered neuronal cytoplasmic tau immunoreactivity and neuropil threads in each low power (×10 microscope objective) field; Grade 2, a mild number of neurofibrillary tangles and neuropil threads in each low power field; Grade 3, a moderate number of neurofibrillary tangles and neuropil threads in each low power field; Grade 4, many densely packed neurofibrillary tangles and neuropil threads in each low power field. Representative images are shown in Figure S1.

### 2.5 Quantitative image analysis

Regarding the TL, the region of interest was defined as the cortical gray matter of an entire involved sulcus and at least one side of the adjacent gyral crest where Aβ deposition was present because the distribution of senile plaques can vary within the cortex [13, 24, 38]. Immunostained sections were scanned at a magnification of ×40 over the entire region with a TissueScope LE120 slide scanner (Huron Digital Pathology, Ontario, Canada). All scanned slides were separately imported to Photoshop (version 24.0; Adobe Inc., CA, USA) and one region of interest was cropped from each image and saved as a new image. The new images were pre‐processed by adjusting the contrast to improve the recognition of small diffuse plaques and reduce the background noise of non‐specific immunoreactivity.

For the striatum, the region of interest was delineated based on anatomical definitions. According to a previous study [22], 10 image squares were taken within the regions of interest as evenly as possible without bias over the entire area (area per image: 0.7 × 0.5 mm) using a bright field microscope (Nikon Eclipse Ci microscope, Nikon Instruments Inc, Tokyo, Japan) equipped with a DS‐Fi3 microscope camera and NIS‐Elements imaging software. All microphotographs were separately imported to Photoshop and pre‐processed by adjusting the contrast to improve the recognition of small diffuse plaques and reduce the background noise of non‐specific immunoreactivity.

The burden of Aβ deposition in each image was quantified using ImageJ/Fiji (ImageJ Version 1.53q) [39] as described previously [13]. This method allows avoiding potential technical bias due to different fixation and enzymatic diaminobenzidine reactions (i.e., the labeling intensity) [13, 40].

Disease category grouping of the cases was performed by one neuropathologist (G.G.K.) based on clinical information and current immunohistochemistry‐based neuropathological criteria. The genotypes for *APOE* and *MAPT* were obtained as described previously [41]. The semiquantitative case selection and quantitative Aβ burden analysis were performed separately by another neuropathologist (S.I.). For the Aβ burden analysis processes, all assessments were performed blinded to clinical and neuropathological diagnoses.

### 2.6 Statistical analysis

Data were analyzed using IBM SPSS Statistics (version 28.0.0.0; SPSS Inc, Chicago, IL, USA), with a significance level set at *p* < 0.05. Fisher's exact test was used for categorical variables (sex and pathological findings). Mann–Whitney *U* test was used for comparing continuous variables (age at death and Aβ deposition burden) between two groups, while Kruskal–Wallis test with post hoc Bonferroni correction was used for comparing three or more groups. Spearman rank correlation coefficient test was used to analyze the relationship between the CGP pathological grading and the number of *APOE* ε4 alleles (*APOE* ε4 score; *n* = 0–2) or the CAA pathological grading. An ordinal regression analysis (ORA) was used to adjust for the effect of age and the *APOE* ε4 score on the Aβ pathological grading for semiquantitative analysis results. For quantitative analysis results, analysis of covariance (ANCOVA) was used to adjust for the effect of age and the *APOE* ε4 score on the Aβ pathological grading. Furthermore, using JMP Pro (version 11.2.0; SAS Institute Inc., Cary, NC, USA), we conducted Ward's hierarchical cluster analysis based on the quantitative results of each Aβ peptide deposition burden.

## 3 RESULTS

### 3.1 NDD group characteristics

Table 1 presents a summary of the cases in the four groups. The mean age at death in the MSA group was significantly lower than that in the LBD group (*p* < 0.01). Additionally, females were older than males in all disease groups, but the differences were not statistically significant (*p* = 0.08/0.48/0.09/0.96 in the LBD/MSA/FTLD‐TDP/PSP groups, respectively). *APOE* ε4 allele frequency was significantly higher in the LBD group than in the FTLD‐TDP and PSP groups (*p* = 0.03, <0.01, respectively), while *MAPT* H1 allele frequency was significantly higher in the PSP group than in the LBD group (*p* < 0.01). In the LBD group, 33 out of 51 cases (67%) showed intermediate or higher ADNC levels, which was significantly higher than in the other groups (vs. all groups; *p* < 0.01). In the LBD group, females had significantly more ADNC‐high cases than males (*p* = 0.01). After adjusting for age, this difference retained its significance (*p* < 0.01; binomial logistic RA). Although Aβ deposition was observed in more than half of the patients in all groups, the positivity was significantly higher in the LBD group than in the other groups (vs. the MSA/FTLD‐TDP/PSP groups; *p* < 0.01/<0.01/0.01). Moreover, the positivity of striatal Aβ deposition was significantly higher in the LBD group than in the other groups (vs. all groups; *p* < 0.01). After selecting cases based on the neuropathological evaluation described above, we excluded the MSA and FTLD‐TDP groups because only one and four cases, respectively, were suitable for further detailed histopathological evaluation.

A summary of the cases that underwent detailed histopathological evaluation, including the evaluation of various Aβ peptides, is shown in Table 3. In the LBD group, the dementia status was available for 31 of 36 patients, and all of them had a clinical diagnosis of dementia. Furthermore, 32 out of 36 cases (89%) exhibited moderate or higher levels of AD co‐pathology. *APOE* genotypes were available for 34 cases in this group, and 29 cases (85%) had one or more *APOE* ε4 allele(s) (7 cases, 21%, were homozygous). Additionally, all cases that showed CGP formation in the TL had at least one *APOE* ε4 allele, and the severity of CGP pathology was significantly correlated with the *APOE* ε4 score (*R* = 0.59, *p* < 0.01; Figure 2C). The effect of the *APOE* ε4 score on the CGP severity remained significant after adjusting for age and sex (*p* < 0.01; ANCOVA). Furthermore, the severity of CGP pathology correlated with the severity of CAA pathology, evaluated using immunostaining for 6F/3D (*R* = 0.76, *p* < 0.01). In the PSP group, seven out of 10 cases (70%) exhibited moderate or higher levels of AD co‐pathology. *APOE* allele status was available for nine cases, seven of which (78%) had one *APOE* ε4 allele. Notably, none of the cases were homozygous for *APOE* ε4 allele in this group, and only one case (10%) showed CGP formation. Seven cases in the LBD group and two cases in the PSP group exhibited Type 1 CAA lesions. Among these, seven out of eight cases (88%) genotyped for *APOE* had one or more *APOE* ε4 allele(s).

**TABLE 3:** Comparison of general pathological findings of suitable cases for detailed histopathological evaluation.

		All	Female	Male	*p* value^a^
LBD cases: SQ only group (F, 19; M, 17); SQ + Q group (F, 18; M, 12)					
Mean age (range)	SQ	76.4 ± 8.7 (56–94)	77.9 ± 10.1 (59–94)	74.8 ± 6.3 (56–85)	0.24
	Q	75.5 ± 8.8 (56–92)	77.0 ± 9.6 (59–92)	73.3 ± 6.7 (56–85)	0.22
*APOE* and *MAPT* allele frequency (%)	#	34 (94)	19 (100)	15 (88)	
	ε2	1 (1)	0	1 (3)	0.44
	ε3	31 (46)	17 (45)	14 (47)	1.00
	ε4	36 (53)	21 (55)	15 (50)	0.81
	H1	48 (71)	24 (63)	24 (80)	0.18
	H2	20 (29)	14 (37)	6 (20)	
LPC category [Lim/Neo; (%)]	SQ	9/27 (25/75)	7/12 (37/63)	2/15 (12/88)	0.13
	Q	8/22 (27/73)	7/11 (39/61))	1/11 (9/91)	0.10
A score [A2/A3] (mean ± SD)	SQ	16/20 (2.6 ± 0.5)	6/13 (2.7 ± 0.5)	10/7 (2.4 ± 0.5)	0.17
	Q	12/18 (2.6 ± 0.5)	5/13 (2.7 ± 0.4)	7/5 (2.4 ± 0.5)	0.17
B score [B1/B2/B3] (mean ± SD)	SQ	4/13/19 (2.4 ± 0.7)	1/5/13 (2.6 ± 0.6)	3/8/6 (2.2 ± 0.7)	0.07
	Q	4/7/19 (2.5 ± 0.7)	1/4/13 (2.7 ± 0.6)	3/3/6 (2.3 ± 0.8)	0.23
C score [C1/C2/C3] (mean ± SD)	SQ	2/20/14 (2.3 ± 0.6)	1/8/10 (2.5 ± 0.6)	1/12/4 (2.2 ± 0.5)	0.16
	Q	1/15/14 (2.4 ± 0.6)	1/7/10 (2.5 ± 0.6)	0/8/4 (2.3 ± 0.5)	0.42
CAA‐type 1/2 (%)^b^		7/19 (19/53)	4/10 (21/53)	3/9 (18/53)	1.00/1.00
CGP‐positive (%)^b^		17 (47)	11 (58)	6 (35)	0.20
CGP severity (±SD)		1.1 ± 1.3	1.4 ± 1.4	0.8 ± 1.2	0.21
PSP cases: SQ only group (F, 6; M, 4); SQ + Q group (F, 4; M, 3)					
Mean age^c^ (range)	SQ	81.7 ± 5.1 (74–93)	81.7 ± 6.0 (74–93)	81.7 ± 2.5 (79–85)	0.71
	Q	82.2 ± 5.9 (74–93)	81.8 ± 7.1 (74–93)	83.0 ± 2.0 (81–85)	1.00
*APOE* and *MAPT* allele frequency (%)	#	9 (90)	5 (83)	4 (100)	
	ε2	2 (11)	1 (10)	1 (13)	1.00
	ε3	9 (50)	4 (40)	5 (63)	0.40
	ε4	7 (39)	5 (50)	2 (25)	0.37
	H1	17 (94)	10 (100)	7 (88)	0.44
	H2	1 (6)	0	1 (12)	
A score [A2/A3] (mean ± SD)	SQ	5/5 (2.5 ± 0.5)	3/3 (2.5 ± 0.5)	2/2 (2.5 ± 0.5)	1.00
	Q	4/3 (2.4 ± 0.5)	2/2 (2.5 ± 0.5)	2/1 (2.3 ± 0.5)	0.86
B score [B1/B2/B3] (mean ± SD)	SQ	3/6/1 (1.8 ± 0.6)	2/3/1 (1.8 ± 0.7)	1/3/0 (1.8 ± 0.4)	0.91
	Q	0/6/1 (2.1 ± 0.3)	0/3/1 (2.3 ± 0.4)	0/3/0 (2.0 ± 0.0)	0.63
C score [C1/C2/C3] (mean ± SD)	SQ	1/7/2 (2.1 ± 0.5)	0/5/1 (2.2 ± 0.4)	1/2/1 (2.0 ± 0.7)	0.76
	Q	1/4/2 (2.1 ± 0.6)	0/3/1 (2.3 ± 0.4)	1/1/1/ (2.0 ± 0.8)	0.86
CAA‐type 1/2 (%)^b^		2/6 (20/60)	0/5 (0/83)	2/1 (50/25)	0.13/0.19
CGP‐pos (%)^b^		1 (10)	0 (0)	1 (10)	0.40

Abbreviations: CAA, cerebral amyloid angiopathy; CGP, coarse‐grained plaque; F, female; Lim, Limbic category; M, male; Neo, neocortical category; Q, quantitative evaluation; SQ, semiquantitative evaluation.

^a^ Females vs. males; Mann–Whitney *U* test or Fischer's exact test.

^b^ The type of CAA and presence or absence of the CGP in the temporal lobe were evaluated using specimens immunostained for Aβ (6F/3D).

^c^ Age at death was not available in one male case.

### 3.2 Evaluation of the neocortical Aβ and tau deposition severity in the LBD and PSP groups

**FIGURE 3:**
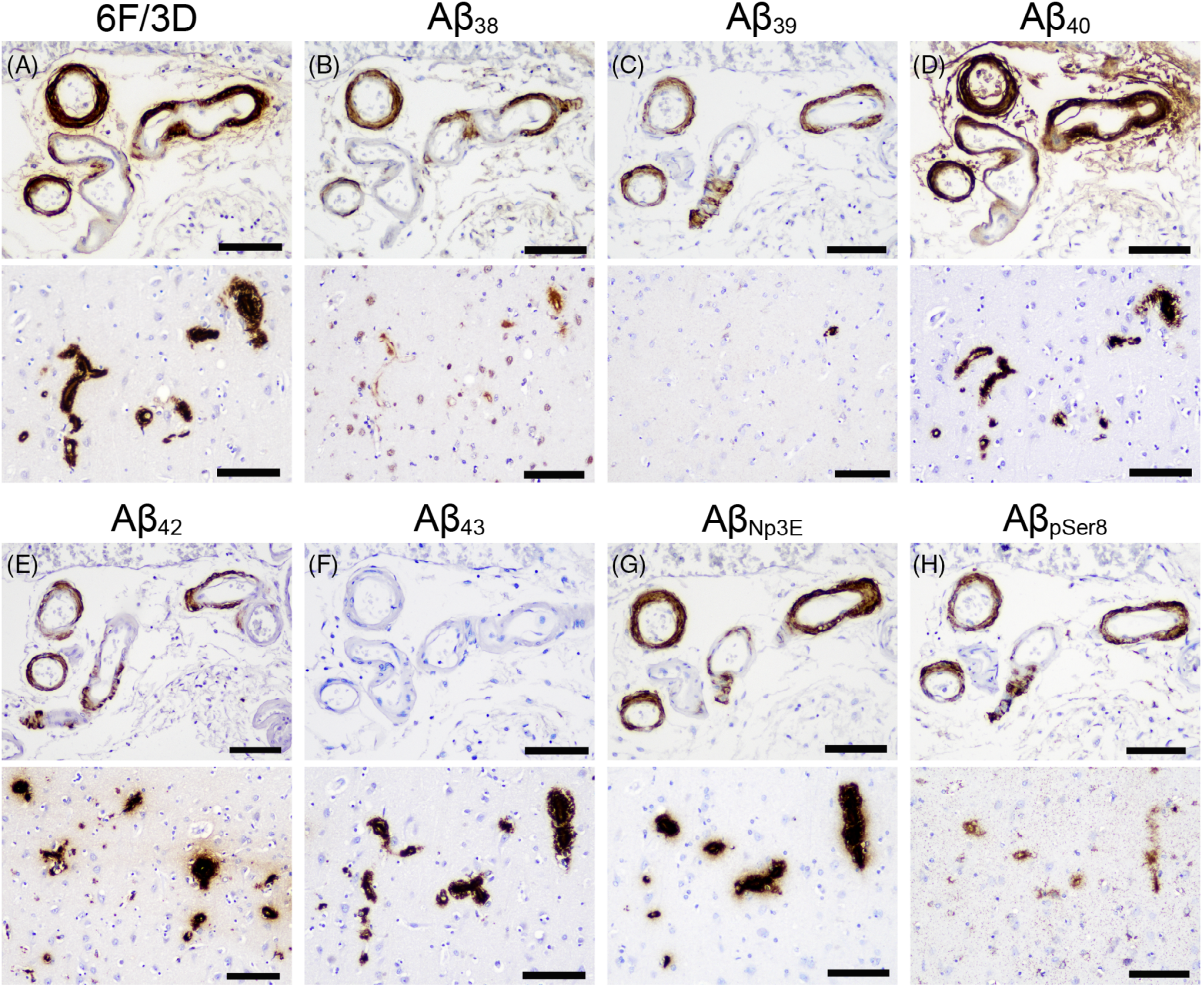
Representative microphotographs of parenchymal Aβ pathology in the temporal lobe. (A–H) LBD case 36. The upper panel of each microphotograph is a low‐magnification image of the specimen, and the lower panel is a high‐magnification view of the sulcal depth. Note that many coarse‐grained plaques, which are strongly positive for 6F/3D, Aβ_40_, and Aβ_Np3E_, are observed in the depth of the sulcus. The region surrounded by the black line indicates the analyzed area. Scale bar = 5 mm (A–H, upper panel), 100 μm (A–H, lower panel).

**FIGURE 4:**
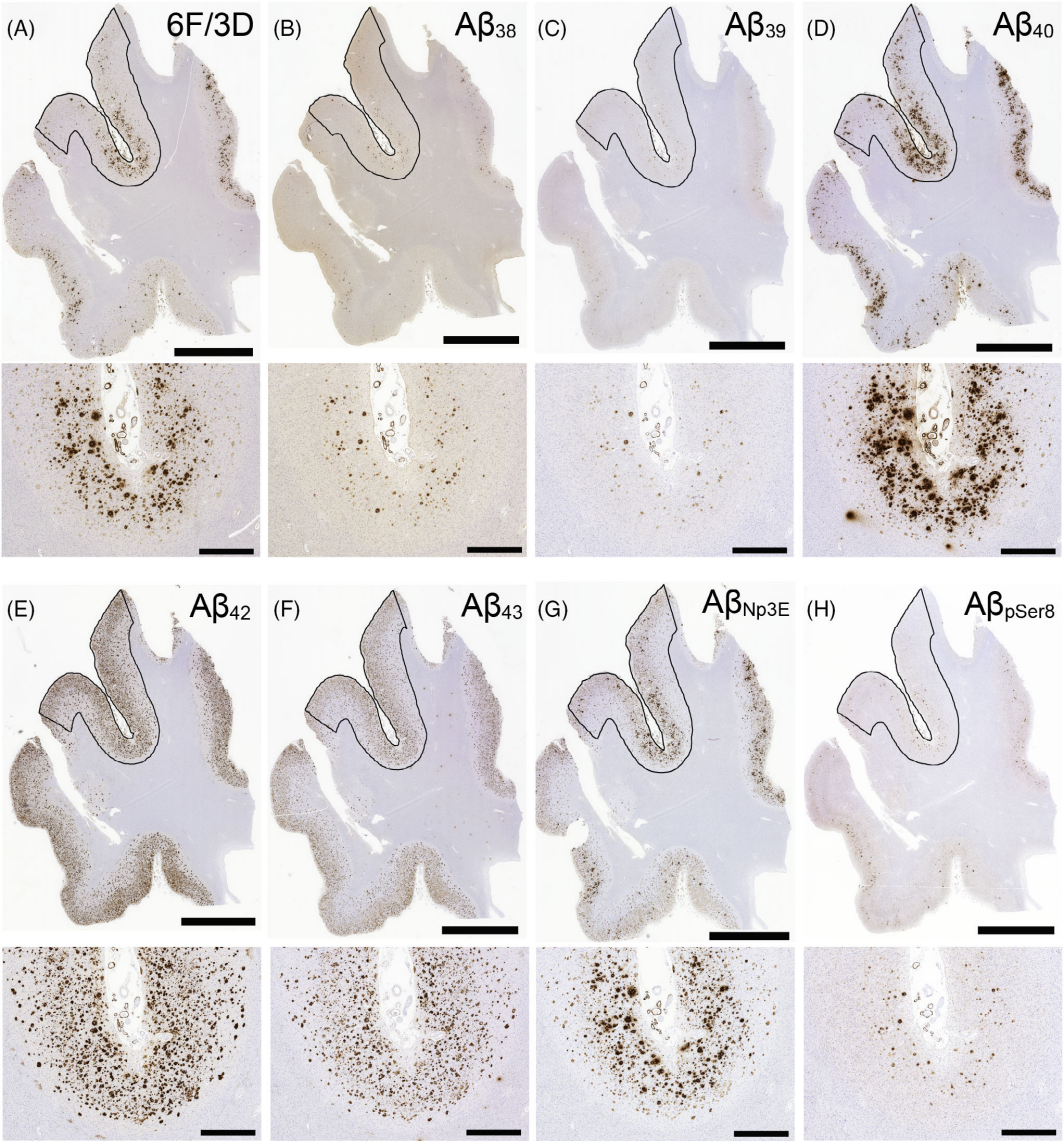
Representative microphotographs of vascular Aβ pathology in the temporal lobe. (A–H) LBD case 20. The upper panels of each microphotograph are typical Type 2 cerebral amyloid angiopathy (CAA) lesions (non‐capillary type CAA), and the lower panels are typical Type 1 CAA lesions (capillary type CAA). Type 2 lesions are strongly positive for 6F/3D, Aβ40, and AβNp3E; moderately positive for Aβ_38_, Aβ_39_, Aβ_42_, and Aβ_pSer8_; and almost negative for Aβ_43_. In contrast, Type 1 lesions are strongly positive for not only 6F/3D, Aβ_40_, and Aβ_Np3E_ but also for Aβ_42_ and Aβ_43_. The immunoreactivity of Aβ_38_, Aβ_39_, and Aβ_pSer8_ in Type 1 lesions is weaker than that in Type 2 lesions. Scale bar = 100 μm.

The representative histological photographs of Aβ plaques and the analysis region are presented in Figure 3, and the representative histological microphotographs of CAA lesions, including Type 1 and 2 lesions, are displayed in Figure 4. A summary of the semi‐quantitative and quantitative analyses results is presented in Table 4, and all analysis results are shown in Table S1. Based on subjective histopathological observations in both disease groups, the amount of neocortical Aβ deposits immunolabeled by Aβ_42_ was the highest (Figure 3E), and the parenchymal deposition of shorter peptides (Aβ_38_ and Aβ_39_) and Aβ_pSer8_ was relatively mild compared to others (Figure 3A–H). Type 2 CAA lesions showed the most robust immunoreactivity for Aβ_40_ and relatively weak immunoreactivity for longer Aβ peptides (Aβ_42_ and Aβ_43_) (Figure 4A–H, upper panels). In contrast, in Type 1 lesions, the immunoreactivity was stronger for these longer peptides than for the shorter peptides (Aβ_38_ and Aβ_39_) (Figure 4A–H, lower panels). The immunoreactivity of Aβ_Np3E_ and Aβ_pSer8_ was strong and intermediate in both types of CAA lesions, respectively (Figure 4G,H). Regarding the semiquantitative severity of CAA, there were no significant differences between the two groups in all Aβ peptides investigated. Considering the extent of deposition and background non‐specific immunoreactivity, we conducted a quantitative analysis on sections immunostained for 6F/3D, Aβ_40_, Aβ_42_, Aβ_43_, and Aβ_Np3E_.

**TABLE 4:** Summary of the semiquantitative (SQ) and quantitative (Q) analysis results in the temporal lobe.

Lewy body disease cases (no. of cases in SQ/Q analysis)										
Anti‐Aβ	All (36/30 cases)			Females (19/18 cases)			Males (17/12 cases)			*p* value^a^
	SQ‐SP	SQ‐VP	Q (range)	SQ‐SP	SQ‐VP	Q (range)	SQ‐SP	SQ‐VP	Q (range)	
6F/3D	2.7 ± 0.4	2.0 ± 1.4	3.6 ± 1.8 (0.9–8.1)	2.9 ± 0.3	2.2 ± 1.4	4.2 ± 1.9 (1.7–8.1)	2.5 ± 0.5	1.8 ± 1.4	2.7 ± 1.3 (0.9–5.5)	0.06/0.42/ **0.03**
Aβ_38_	1.3 ± 0.5	1.6 ± 1.3	NE	1.2 ± 0.4	1.9 ± 1.3	NE	1.4 ± 0.6	1.4 ± 1.3	NE	0.43/0.24/NE
Aβ_39_	1.1 ± 0.4	1.6 ± 1.3	NE	1.1 ± 0.3	1.8 ± 1.3	NE	1.1 ± 0.4	1.4 ± 1.2	NE	0.85/0.42/NE
Aβ_40_	2.5 ± 0.6	2.1 ± 1.4	2.7 ± 2.2 (0.2–11.4)	2.7 ± 0.7	2.2 ± 1.4	3.0 ± 2.6 (0.2–11.4)	2.4 ± 0.5	1.9 ± 1.4	2.1 ± 1.0 (0.9–3.8)	0.11/0.62/0.55
Aβ_42_	3.7 ± 0.5	1.8 ± 1.4	11.2 ± 3.9 (2.1–23.1)	3.9 ± 0.4	1.9 ± 1.4	12.3 ± 4.4 (2.1–23.1)	3.5 ± 0.5	1.6 ± 1.3	9.7 ± 2.1 (6.3–13.3)	**0.02** /0.55/0.05
Aβ_43_	3.1 ± 0.5	1.1 ± 1.1	5.5 ± 3.2 (1.7–16.9)	3.4 ± 0.5	1.1 ± 1.0	6.5 ± 3.7 (3.0–16.9)	2.8 ± 0.4	1.2 ± 1.2	3.9 ± 1.2 (1.7–6.2)	**<0.01** ^b^/0.75/**0.03**
Aβ_Np3E_	2.8 ± 0.5	1.9 ± 1.3	4.1 ± 2.3 (1.4–10.1)	2.9 ± 0.6	2.1 ± 1.3	4.7 ± 2.5 (1.8–10.1)	2.6 ± 0.5	1.6 ± 1.3	3.3 ± 1.6 (1.4–7.1)	0.19/0.45/0.10
Aβ_pSer8_	1.4 ± 0.6	1.4 ± 1.2	NE	1.3 ± 0.6	1.5 ± 1.2	NE	1.4 ± 0.6	1.3 ± 1.2	NE	0.35/0.64/NE
Anti‐p‐tau	SQ (total p‐tau IR)			SQ (total p‐tau IR)			SQ (total p‐tau IR)			*p* value^c^
AT8	2.6 ± 1.1			3.1 ± 0.9			2.1 ± 1.1			**0.02**

Progressive supranuclear palsy cases (no. of cases in SQ/Q analysis)										
Anti‐Aβ	All (10/7 cases)			Females (6/4 cases)			Males (4/3 cases)			*p* value^a^
	SQ‐SP	SQ‐VP	Q (range)	SQ‐SP	SQ‐VP	Q (range)	SQ‐SP	SQ‐VP	Q (range)	
6F/3D	2.2 ± 0.4	1.7 ± 1.0	1.8 ± 0.9 (0.7–3.5)	2.2 ± 0.4	1.7 ± 0.9	2.0 ± 0.9 (1.1–3.5)	2.3 ± 0.4	1.8 ± 1.1	1.5 ± 0.8 (0.7–2.6)	1.00/1.00/0.63
Aβ_38_	1.0 ± 0.4	1.6 ± 0.9	NE	1.2 ± 0.4	1.5 ± 1.0	NE	0.8 ± 0.4	1.8 ± 0.8	NE	0.35/0.76/NE
Aβ_39_	0.9 ± 0.3	1.4 ± 1.0	NE	1.0 ± 0.0	1.0 ± 1.0	NE	0.8 ± 0.4	2.0 ± 0.7	NE	0.61/0.17/NE
Aβ_40_	1.9 ± 0.3	1.9 ± 1.1	0.7 ± 0.2 (0.4–1.2)	2.0 ± 0.0	2.0 ± 1.2	0.7 ± 0.3 (0.4–1.2)	1.8 ± 0.4	1.8 ± 1.1	0.6 ± 0.1 (0.5–3.7)	0.61/0.91/0.63
Aβ_42_	3.1 ± 0.3	1.7 ± 1.0	8.1 ± 4.4 (2.4–18.0)	3.2 ± 0.4	1.7 ± 0.9	10.4 ± 4.4 (7.1–18.0)	3.0 ± 0.0	1.8 ± 1.1	5.0 ± 1.9 (2.4–6.6)	0.76/1.00/0.06
Aβ_43_	2.6 ± 0.5	1.1 ± 1.0	2.3 ± 1.2 (1.3–5.2)	2.7 ± 0.5	1.0 ± 1.2	2.0 ± 0.1 (1.8–2.1)	2.5 ± 1.3	1.3 ± 0.8	2.6 ± 1.8 (1.3–5.2)	0.76/0.76/0.63
Aβ_Np3E_	2.8 ± 0.4	1.6 ± 1.1	2.8 ± 1.0 (1.0–4.1)	2.8 ± 0.4	1.5 ± 1.1	2.7 ± 0.3 (2.3–3.1)	2.8 ± 0.4	1.8 ± 1.1	3.0 ± 1.4 (1.0–4.1)	0.91/0.76/0.63
Aβ_pSer8_	1.4 ± 0.5	1.4 ± 1.0	NE	1.5 ± 0.5	1.3 ± 0.9	NE	1.3 ± 0.4	1.5 ± 1.1	NE	0.61/1.00/NE
Anti‐p‐tau	SQ (total p‐tau IR)			SQ (total p‐tau IR)			SQ (total p‐tau IR)			*p* value^c^
AT8	1.9 ± 1.0			2.0 ± 1.2			1.8 ± 0.8			0.91

*Note*: Boldface signifies values that are significant at *p* < 0.05. Underlined and bold values are those where the *p* value remained significant after correction for age at death and the *APOE* ε4 score.

Abbreviations: IR, immunoreactivity; NE, not evaluated; SP, senile plaque pathology grading; VP, vascular pathology grading.

^a^ Comparison of the SQ‐SP/SQ‐VP/Q values between females and males (Mann–Whitney *U* test).

^b^ We could not adjust the value due to data separation (ordinal regression analysis).

^c^ Comparison of the SQ value between females and males (Mann–Whitney *U* test).

In the LBD group, females showed more severe Aβ plaque pathology than males in most of Aβ‐immunostainings investigated. The statistical significance was reached for semiquantitative grading of Aβ_42_ and Aβ_43_, as well as for the quantitative burden of pan‐Aβ (6F/3D) and Aβ_43_. Notably, only males displayed mild Aβ_43_ deposition (G2), while severe Aβ_43_ deposition (G4) was observed exclusively in females (Table S1). After adjusting for age and the *APOE* ε4 score, the differences remained statistically significant for semiquantitative grading of Aβ_42_ (*p* = 0.02; ORA) and quantitative burden of 6F/3D (*p* = 0.04; ANCOVA). Additionally, although the observed difference did not reach statistical significance, there was a tendency for females to exhibit a higher quantitative Aβ_43_ deposition burden compared to males, following adjustment (*p* = 0.054; ANCOVA). Similar levels of Aβ_38_, Aβ_39_, and Aβ_pSer8_ deposition were observed between sexes. Furthermore, no significant differences were found in the severity of CAA pathological grading between males and females. Regarding p‐tau pathology, females showed significantly more severe tau deposition than males. After adjusting for age and the *APOE* ε4 score, the difference remained statistically significant (*p* = 0.03; ORA). We additionally conducted an analysis to explore the impact of the *APOE* ε4 score on the quantitative burden of Aβ deposition. The findings are summarized in Table 5. Out of the 30 cases included in the quantitative analysis, four cases were classified as Group 0 (score of 0), 19 cases as Group 1 (score of 1), and seven cases as Group 2 (score of 2). Group 2 exhibited higher quantitative Aβ deposition burdens across all peptides investigated compared to the other groups, with significant differences observed for Aβ_42_. Moreover, there was a positive correlation between the *APOE* ε4 score and the quantitative deposition burden of all investigated Aβ peptides in this cohort, with significant correlations found for Aβ_40_, Aβ_42_, and Aβ_Np3E_.

**TABLE 5:** Summary of clinical information and analysis results for each *APOE* ε4 score group within the LBD cohort.^a^

	Group 0	Group 1	Group 2	*p* value	*R*/*p* value^c^
				All (G0‐1/G0‐2/G1‐2)^b^	
No. of cases	4	19	7		
Mean age (range)	72.5 ± 8.3 (62–85)	76.4 ± 8.3 (59–92)	75.0 ± 9.9 (56–88)	0.678	
Sex (F/M)	1/3	13/6	4/3		
6F/3D (range)	2.5 ± 0.6 (1.6–3.0)	3.5 ± 1.9 (0.9–8.1)	4.6 ± 1.7 (2.4–8.0)	0.15	0.36/0.05
Aβ_40_ (range)	1.5 ± 0.2 (1.2–1.8)	2.7 ± 2.6 (0.2–11.4)	3.4 ± 1.4 (1.2–5.4)	0.11	0.40/**0.03**
Aβ_42_ (range)	8.3 ± 1.1 (6.8–9.4)	11.3 ± 4.3 (2.1–23.1)	12.8 ± 2.6 (9.3–17.0)	**0.03** (0.12/**0.02**/0.58)	0.48/<**0.01**
Aβ_43_ (range)	3.8 ± 0.4 (3.2–4.4)	5.4 ± 3.6 (1.7–16.9)	6.5 ± 2.7 (3.0–11.9)	0.19	0.34/0.07
Aβ_Np3E_ (range)	2.7 ± 0.6 (2.1–3.4)	3.9 ± 2.3 (1.4–10.1)	5.5 ± 2.2 (2.2–8.6)	0.08	0.42/**0.02**

*Note*: Boldface signifies values that are significant at *p* < 0.05.

Abbreviations: G, Group; LBD, Lewy body disease.

^a^ Group 0 comprises individuals who are not carriers of *APOE* ε4 allele (*APOE* score 0), Group 1 includes cases of heterozygosity for *APOE* ε4 allele (*APOE* score 1), and Group 2 includes cases of homozygosity for *APOE* ε4 allele (*APOE* score 2).

^b^ Kruskal–Wallis test with post hoc Bonferroni correction.

^c^ Correlation between the *APOE* ε4 score and the quantitative Aβ deposition burden in the temporal lobe (Spearman rank correlation coefficient test).

In the PSP group, the mean values of all Aβ peptides subjected to quantitative analysis were lower compared to the LBD group, with significant differences observed for 6F/3D, Aβ_40_, Aβ_42_, and Aβ_43_ (*p* < 0.01, <0.01, 0.02, <0.01, respectively). While females exhibited a mean Aβ_42_ deposition burden more than double that of males, the difference did not attain statistical significance. Unlike the LBD group, there were no notable differences in Aβ pathology between females and males. In terms of p‐tau pathology, there was no significant difference observed between the sexes.

### 3.3 Evaluation of the striatal Aβ deposition severity in the AD, LBD, and PSP groups

**FIGURE 5:**
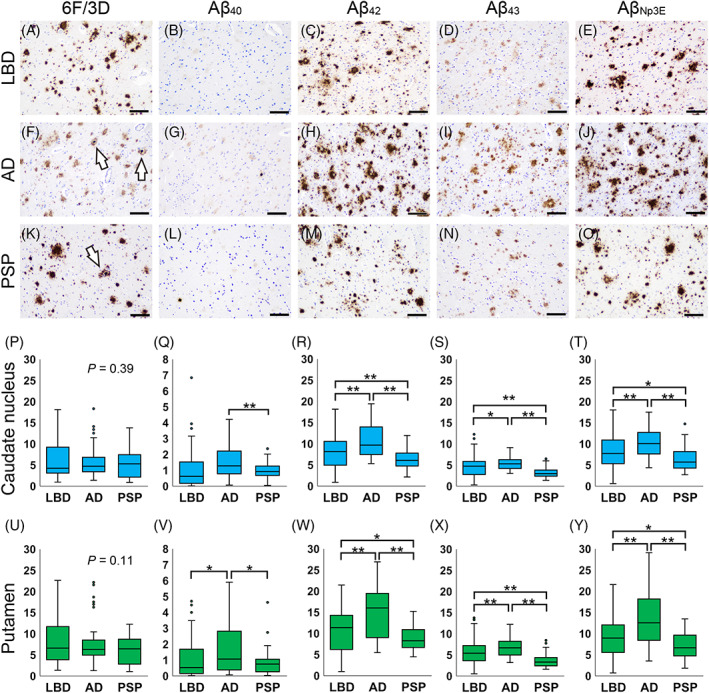
Representative microphotographs of parenchymal Aβ pathology and results of the quantitative Aβ deposition analysis in the striatum. (A–E) Lewy body disease case (LBD6); (F–J) Alzheimer's disease case (AD5); (K–O) progressive supranuclear palsy case (PSP1). (P–T) Summaries of the results in the caudate nucleus (Caudate N). (U–Y) Summaries of the results in the putamen. Representative microphotographs/results of the quantitative analysis of specimens immunostained for 6F/3D (A, F, K/P, U); Aβ_40_ (B, G, L/Q, V); Aβ_42_ (C, H, M/R, W); Aβ_43_ (D, I, N/S, X); Aβ_Np3E_ (E, J, O/T, Y). Arrows indicate cored plaques. (Q, V) Note that the amount of Aβ_40_ deposition is lower than that of the other Aβ peptides. *p < 0.05; **p < 0.01 (Kruskal–Wallis test with post hoc Bonferroni correction). Scale bar = 100 μm.

Figure 5 exhibits representative histological microphotographs and analysis results of the three disease groups. The representative histological microphotographs of Aβ plaques immunolabeled by Aβ_pSer8_ are presented in Figure S2, a summary of the clinical information and the results from both the semiquantitative and quantitative analyses in Table 6 and all the analysis findings in Table S2. Based on the criteria detailed above only 15 LBD, 6 AD, and 5 PSP cases were selected for detailed analysis (i.e., those showing G3 or above severity of striatal Aβ deposition; see Figure 1 for case selection details). Most Aβ plaques displayed diffuse‐type morphology (Figure 5A–O). There were no significant differences in the severity of Aβ deposition labeled by antibody 6F3D among the three groups in the semiquantitative analysis. Although not statistically significant (*p* = 0.07, see Table 6), cored plaque formation tended to be greater in the AD and PSP groups and less in the LBD group (Figure 5A,F,K). Similar to the TL, striatal Aβ deposits mainly consisted of Aβ_42_ (Figure 5C,H,M), and Aβ_38_ and Aβ_39_ deposition was minimal. In contrast, there was less deposition of Aβ_40_ (Figure 5B,G,L) in the striatum. The AD and PSP groups had higher Aβ_pSer8_ deposition in both the CN and putamen than the LBD group, with significant differences between the three groups but no significant differences in either combination between the two groups. Given the amount of deposition and the background immunoreactivity, only sections immunostained for 6F/3D, Aβ_40_, Aβ_42_, Aβ_43_, and Aβ_Np3E_ were analyzed quantitatively. In immunohistochemistry for 6F/3D, there were no statistically significant differences among the three groups both in the CN and putamen (Figure 5P,U). Furthermore, while the AD group showed significantly higher Aβ_40_ deposition in the CN and putamen than the other groups (Figure 5Q,V), the amount of deposition was low in all groups. In contrast, the AD group showed significantly higher Aβ_42_ (Figure 5R,W), Aβ_43_ (Figure 5S,X), and Aβ_Np3E_ (Figure 5T,Y) deposition in the CN and putamen than the other groups. To rigorously investigate these differences, we further compared the burden of Aβ deposition among only individuals with ADNC‐A score of 3 in the three groups. The data are provided in Table S3 and Figure S3. In comparing the AD and LBD groups, only the deposition burden of Aβ_42_ in the putamen was significantly more severe in the AD group (*p* = 0.02). In contrast, when comparing the AD and PSP groups, 6F/3D deposition was significantly more severe in the PSP group in both the CN and putamen (*p* < 0.01, 0.04, respectively), whereas the deposition burden of Aβ_42_, Aβ_43_, and Aβ_Np3E_ were significantly more severe in the AD group in both the CN and putamen (Aβ_42_, *p* < 0.01 in both; Aβ_43_, *p* = 0.03, < 0.01; Aβ_Np3E_, *p* < 0.01 in both, respectively). Due to the unavailability of *APOE* genotypes for all AD cases in this cohort, a comparative evaluation of the impact of *APOE* score among the three groups could not be performed. However, no significant difference was observed between the LBD and PSP groups (*p* = 1.00; Mann–Whitney *U* test). Furthermore, we compared the burden of Aβ deposition among only individuals with ADNC‐A score of 2. In this cohort, only cases in the LBD and PSP groups were included. The data are provided in Table S4 and Figure S4. Although there were no significant differences in sex ratio or the *APOE* score between the two groups (*p* = 1.00, 0.79, respectively), the deposition burden of 6F/3D and Aβ_43_ was significantly more severe in the LBD group in both the CN and putamen (6F/3D, *p* = 0.02, < 0.01; Aβ_43_, *p* < 0.01 in both, respectively).

**TABLE 6:** Summary of the clinical, pathological, semiquantitative (SQ), and quantitative (Q) results of striatal Aβ analysis.

		LBD	AD	PSP	*p* value^a^
					All (LBD–AD/LBD–PSP/PSP–AD)
No. of cases		15	6	5	
Mean age (range)		75.5 ± 8.1 (59–90)	77.2 ± 5.4 (66–82)	78.8 ± 3.1 (74–82)	0.73
Sex (F/M)		9/6	4/2	3/2	
ADNC A score (A2/A3)		6/9	0/6	3/2	
CP grade (0/1/2/3)^b^		5/9/1/0	1/2/2/1	0/3/2/0	0.07
6F/3D	SQ‐CN	3.3 ± 0.5	3.2 ± 0.4	3.4 ± 0.5	0.68
	SQ‐Put	3.5 ± 0.5	3.5 ± 0.5	3.4 ± 0.5	0.88
	Q‐CN	6.2 ± 4.3 (0.9–18.1)	5.9 ± 3.7 (1.4–18.3)	5.2 ± 3.2 (0.9–13.8)	0.39
	Q‐Put	8.0 ± 5.0 (1.4–22.6)	8.0 ± 5.6 (1.3–22.5)	6.0 ± 3.3 (1.1–12.2)	0.11
Aβ_38_	SQ‐CN	0.0 ± 0.0	0.2 ± 0.4	0.2 ± 0.4	0.24
	SQ‐Put	0.1 ± 0.2	0.2 ± 0.4	0.0 ± 0.0	0.58
Aβ_39_	SQ‐CN	0.1 ± 0.3	0.0 ± 0.0	0.0 ± 0.0	0.47
	SQ‐Put	0.0 ± 0.0	0.0 ± 0.0	0.0 ± 0.0	1.00
Aβ_40_	SQ‐CN	1.8 ± 0.8	2.2 ± 0.7	2.0 ± 0.0	0.54
	SQ‐Put	1.8 ± 0.7	2.2 ± 0.7	1.6 ± 0.5	0.43
	Q‐CN	1.0 ± 1.0 (0.0–6.8)	1.5 ± 1.1 (0.1–4.2)	1.0 ± 0.5 (0.0–2.4)	**<0.01** (**<0.01**/0.50/0.16)
	Q‐Put	1.0 ± 1.1 (0.0–4.7)	1.6 ± 1.5 (0.1–5.9)	0.8 ± 0.8 (0.0–4.6)	**<0.01** (**0.01**/1/**0.02**)
Aβ_42_	SQ‐CN	3.6 ± 0.5	4.0 ± 0.0	3.2 ± 0.4	**0.03** (0.29/0.36/**0.02**)
	SQ‐Put	3.7 ± 0.4	4.0 ± 0.0	3.8 ± 0.4	0.39
	Q‐CN	8.2 ± 3.9 (0.9–18.1)	11.0 ± 4.0 (5.3–19.4)	6.5 ± 2.5 (2.2–11.9)	**<0.01** (**<0.01**/**<0.01**/**<0.01**)
	Q‐Put	10.6 ± 4.8 (1.0–21.4)	14.8 ± 5.8 (5.4–26.9)	8.6 ± 2.5 (4.5–15.2)	**<0.01** (**<0.01**/**0.03**/**<0.01**)
Aβ_43_	SQ‐CN	3.1 ± 0.4	3.2 ± 0.4	3.0 ± 0.0	0.77
	SQ‐Put	3.2 ± 0.5	3.5 ± 0.5	3.0 ± 0.0	0.24
	Q‐CN	4.6 ± 2.3 (0.3–12.3)	5.3 ± 1.4 (3.0–9.1)	3.3 ± 1.3 (1.4–6.8)	**<0.01** (**0.02**/**<0.01**/**<0.01**)
	Q‐Put	5.6 ± 2.8 (0.5–14.0)	6.8 ± 2.2 (3.2–12.2)	3.6 ± 1.6 (1.6–8.4)	**<0.01** (**<0.01**/**<0.01**/**<0.01**)
Aβ_Np3E_	SQ‐CN	3.5 ± 0.6	3.8 ± 0.4	3.4 ± 0.5	0.33
	SQ‐Put	3.5 ± 0.6	3.8 ± 0.4	3.4 ± 0.5	0.33
	Q‐CN	7.9 ± 4.0 (0.6–18.0)	10.2 ± 3.1 (4.4–17.5)	6.5 ± 2.7 (2.7–14.7)	**<0.01** (**<0.01**/**0.03**/**<0.01**)
	Q‐Put	9.2 ± 4.7 (0.7–21.6)	13.8 ± 6.2 (3.5–29.1)	7.1 ± 3.2 (1.8–13.5)	**<0.01** (**<0.01**/**0.02**/**<0.01**)
Aβ_pSer8_	SQ‐CN	1.5 ± 0.6	2.2 ± 0.7	2.4 ± 0.5	**0.03** (0.22/0.06/1.00)
	SQ‐Put	1.4 ± 0.6	2.2 ± 0.7	2.2 ± 0.4	**0.02** (0.08/0.07/1.00)

*Note*: Boldface signifies values that are significant at *p* < 0.05.

Abbreviations: AD, Alzheimer's disease; ADNC, Alzheimer's disease neuropathologic change; CN, caudate nucleus; CP, cored plaque; LBD, Lewy body disease; PSP, progressive supranuclear palsy; Put, putamen; SQ, semiquantitative; Str, striatal.

^a^ Evaluated using the Kruskal–Wallis test with post hoc Bonferroni correction.

^b^ The CERAD score A (sparse) corresponds to score of 1, B (moderate) corresponds to score of 2, and C (frequent) corresponds to score of 3.

### 3.4 Subgroups classified with cluster analysis

**FIGURE 6:**
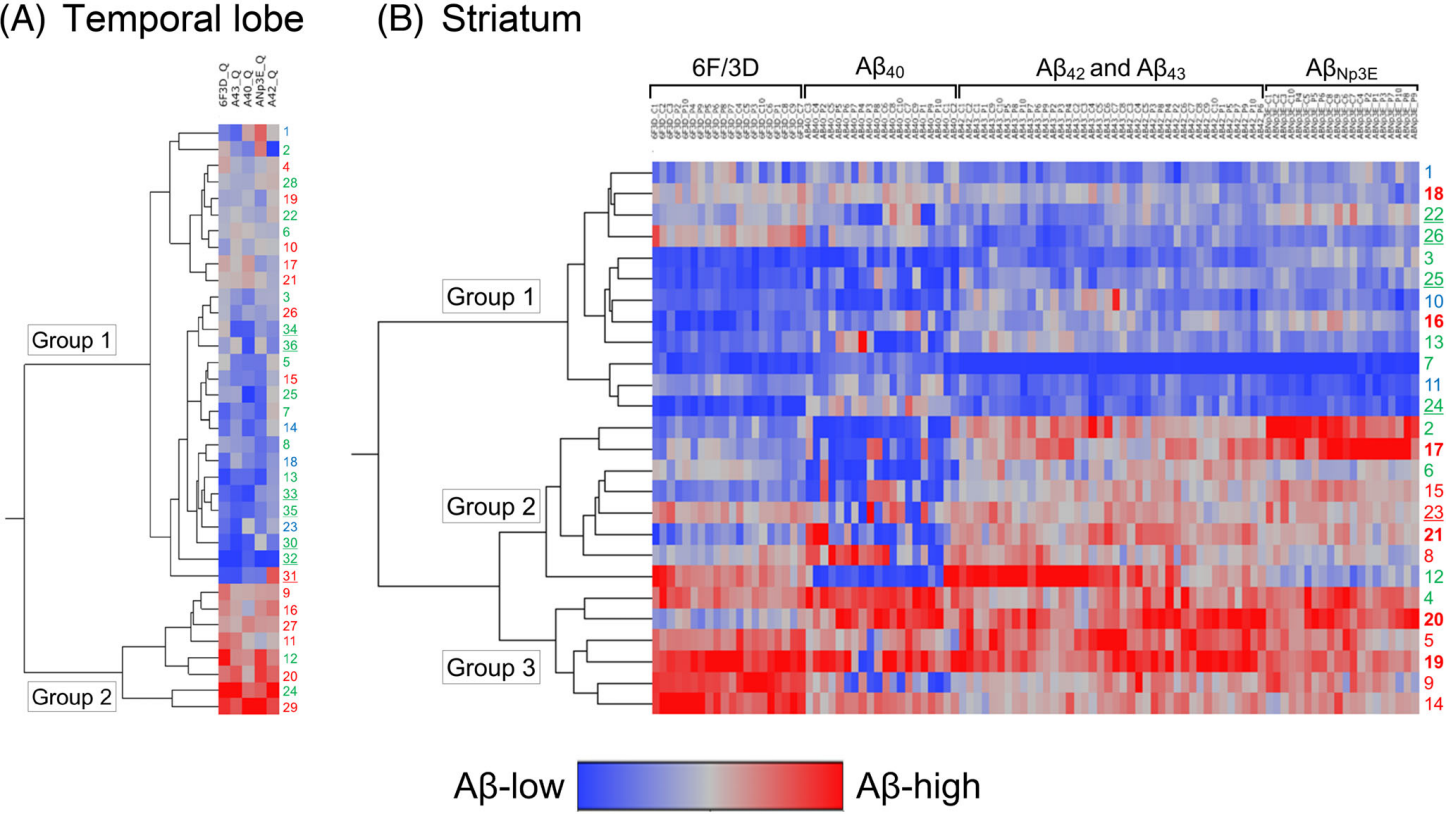
Wards hierarchical cluster analysis using the quantitative Aβ deposition burden. (A) Temporal cortex. (B) Striatum. (A) Individuals in the Lewy body disease (LBD) group (1–29) and progressive supranuclear palsy (PSP) group (30–36). (B) Individuals in the LBD group (1–15), Alzheimer's disease (AD) group (16–21), and PSP group (22–26). Dendrogram of cluster analysis (left panel) and the severity of deposition burden in each Aβ peptide (right panels). Regular typeface: LDB cases; bold typeface: AD cases; underlined typeface: PSP cases. Cases highlighted in blue indicate low Alzheimer's disease neuropathologic change (ADNC) levels, cases highlighted in green indicate intermediate ADNC levels, and cases highlighted in red indicate high ADNC levels.

We conducted a cluster analysis based on the quantitative deposition burden of each Aβ peptide in the TL and striatum (Figure 6). Clinical and pathological information for each group can be found in Tables S5 and S6.

In the TL, the cluster analysis categorized the cases into two groups (Figure 6A). Table S5 provides details for each group. Group 1 (consisting of 28 cases) exhibited lower levels of Aβ deposition compared to Group 2 (consisting of 8 cases). Therefore, Group 1 was designated as the “low Aβ deposition group.” Notably, all PSP cases were classified within this group, supporting the finding of less Aβ deposition compared to LBD cases. Group 2 displayed significantly higher levels of Aβ deposition compared to Group 1 across all investigated Aβ peptides (*p* < 0.01). Hence, Group 2 was identified as the “high Aβ deposition group.” Moreover, Group 2 demonstrated a significantly greater proportion of cases with ADNC‐high in comparison to Group 1 (75% vs. 29%; *p* = 0.04), providing support for the association between tau pathology and the extent of Aβ deposition in the cerebral cortex. Among the eight cases in this group, seven were females. The frequency of *APOE* ε4 allele was significantly higher in Group 2 compared to Group 1 (*p* = 0.046).

In the striatum, the cluster analysis divided the cases into three groups (Figure 6B). Table S6 provides additional information for each group. Group 1 (consisting of 12 cases) exhibited the lowest degree of Aβ deposition among the three groups, particularly with respect to Aβ_42_, Aβ_43_, and Aβ_Np3E_. Consequently, Group 1 was termed the “low Aβ deposition group.” Four out of five PSP cases (all with intermediate ADNC level) were included in this group. Group 2 (consisting of eight cases) displayed significantly higher levels of Aβ deposition compared to Group 1, except for Aβ_40_ (*p* < 0.01). Thus, Group 2 was designated the “Intermediate Aβ deposition group.” This group included one PSP case with a high ADNC level. Group 3 (consisting of six cases) exhibited the most severe Aβ deposition among the three groups, leading to its identification as the “high Aβ deposition group.” No PSP cases were included in this group. Interestingly, cases in the LBD and AD groups were distributed relatively evenly across the three groups, indicating a significant level of heterogeneity among cases within these groups.

## 4 DISCUSSION

This study aimed to investigate the spectrum of AD‐related pathology in four non‐AD NDDs using immunohistochemistry. Our findings revealed several significant observations: (1) there are notable variations in the severity of AD‐related pathology among non‐AD NDDs; (2) the frequency of the *APOE* ε4 allele is higher in the LBD group compared to the PSP group, and the *APOE* ε4 score is associated with the severity of coarse‐grained plaque (CGP), a recently described type of Aβ plaque primarily composed of Aβ_40_ [24]; (3) females in the LBD group exhibit a significantly higher burden of Aβ (6F/3D, Aβ_42_, and Aβ_43_) and p‐tau pathology compared to males; (4) in the LBD group, *APOE* ε4 status is positively correlated with the deposition burden of all investigated Aβ peptides; (5) differences exist in the composition of deposited Aβ peptides between Type 1 and Type 2 CAA lesions; (6) the deposition burden of peptides with high aggregation capacity in the striatum in the AD and LBD groups, especially Aβ_43_, was significantly higher than in the PSP group; (7) the composition of Aβ peptides deposited in the striatum varies considerably from case to case in the AD and LBD groups, but is relatively consistent in the PSP group; and (8) cluster analysis corroborated these observations.

We have demonstrated that the LBD group exhibited the most severe AD‐related pathologies among the four non‐AD NDDs investigated, which aligns with previous neuropathological studies [3, 5]. In contrast, the MSA group, also categorized as an α‐synucleinopathy [42], displayed the mildest AD pathology. One possible reason for this disparity is that the mean age at death in the MSA group was significantly younger than that in the LBD group in this study. However, previous studies have indicated that the incidence of concomitant ADNC in MSA is generally lower than in LBD [1, 16, 43, 44], suggesting that factors beyond age contribute to these differences. There is a growing understanding that the pathological aggregation of one protein can synergistically initiate or promote the aggregation of different species [45]. Synergistic interactions between Aβ and α‐synuclein, tau and α‐synuclein, as well as these three proteins together, have been reported [46, 47, 48, 49, 50]. Given that the strain of α‐synuclein differs in LBD and MSA [51, 52, 53], we can speculate that the interaction between α‐synuclein and AD‐type pathological proteins is weaker in MSA compared to LBD.

Furthermore, it is noteworthy that the severity of Aβ pathology was significantly lower in the PSP group compared to the LBD group, despite no significant difference in mean age at death between the two groups. It is important to mention that the *APOE* ε4 and *MAPT* H1 allele frequencies differ between the two disease groups in our cohort. In particular, there were no cases homozygous for *APOE* ε4 in the PSP group. However, recent studies on large PSP cohorts have consistently demonstrated low levels of ADNC among individuals with PSP [54, 55]. Furthermore, Robinson et al. found that the overall burden of FTLD‐tau, which includes neuronal, astrocytic, and oligodendrocytic tau, did not correlate with Thal phase and CERAD neuritic plaque scores [56]. The properties of deposited tau are known to be different between PSP and AD [57, 58, 59], suggesting that the synergistic interaction between tau and Aβ may be weaker in PSP compared to AD.

Semi‐quantitative and quantitative analyses revealed that females in the LBD group had higher AD‐related pathologies than males. These findings support previous results reported by Bayram et al., who evaluated a similar cohort [14]. Specifically, females in this group exhibited statistically significant higher Aβ deposition, labeled by immunostaining for 6F/3D, Aβ_42_, and Aβ_43_. The 6F/3D antibody is predicted to react primarily with Aβ generated through β‐secretase and γ‐secretase, known as the amyloidogenic pathway [60]. Additionally, both Aβ_42_ and Aβ_43_ are hydrophobic and prone to form toxic aggregates [7, 61, 62]. Hence, it is suggested that females have more neurotoxic Aβ peptides deposited than males in the LBD group. It is well known that approximately two‐thirds of individuals with AD are females, and the female sex is considered a major risk factor for developing AD [10, 17, 63, 64, 65]. Sex hormones have been proposed as a potential explanation for this difference, and several studies have indicated that sex hormones have protective effects against Aβ, such as regulating the production, transport, and removal of Aβ [66, 67, 68], and preventing Aβ toxicity to mitochondria and reducing neuronal vulnerability to apoptosis [69, 70]. Therefore, we can speculate that females with LBD tend to be more predisposed and vulnerable to greater Aβ deposition, and associated tau deposition [71, 72, 73], resulting in differences in the severity of AD pathology between the sexes.

*APOE* ε4 is a significant genetic risk factor for late‐onset AD dementia [64]. As the apoE4 protein is less efficient at clearing Aβ [74], the abnormal accumulation of Aβ protein is considered one of the main causes of increased risk for AD [75]. Interestingly, Dickson et al. reported that *APOE* ε4 is associated with an increased risk of transitional and diffuse LBD in cases with moderate or high AD pathology [76]. In our cohort, approximately 90% of LBD cases with moderate or higher AD levels had at least one *APOE* ε4 allele, corroborating their result. Therefore, it is suggested that *APOE* ε4 plays a crucial role in the pathophysiology of not only AD but also LBD. We can speculate that the deposited Aβ facilitates α‐synuclein deposition analogously to that proposed for neurofibrillary pathology in AD [71, 72, 73, 76].

The CGP is a recently described type of Aβ plaque that mainly consists of Aβ_40_ [24]. In this study, approximately half of the individuals with LBD showed CGPs. It is more common in individuals with early‐onset AD and is associated with an *APOE* ε4 homozygous status and CAA [24]. Consistent with this study [24], we showed that the *APOE* ε4 score and the severity of CAA pathology were significantly correlated with the CGP grading. Vidal et al. have reported two cases of neocortical LBD homozygous for the *APOE* ε4 alleles showing Aβ_40_‐predominant plaques consistent with the CGP [77]. Thus, it is speculated that the formation of CGP in LBD occurs based on a similar pathophysiology as in AD, especially associated with *APOE* ε4. Furthermore, CGPs were only observed in AD cases with clinical dementia [24]. Whether the presence of CGPs contributes independently to the cognitive decline in LBD merits further studies.

We found that Type 2 CAA lesions contain more Aβ peptides with shorter C‐termini (Aβ_38_, Aβ_39_, Aβ_40_), while Type 1 CAA lesions contain more Aβ peptides with longer C‐termini (Aβ_42_, Aβ_43_). These findings are consistent with the results of some previous neuropathological studies that found Aβ_40_ predominated over Aβ_42_ in Type 2 CAA lesions, whereas Aβ_42_ predominated over Aβ_40_ in Type 1 CAA lesion [78, 79]. It is reasonable to hypothesize that Aβ peptides with longer C‐termini are more prone to aggregate and therefore show deposition on more peripherally‐located vessels (capillaries). To the best of our knowledge, this is the first report to comprehensively examine the immunohistochemical composition of deposited Aβ peptides in Type 1 and Type 2 CAA lesions.

The discrepancy in the severity of quantitative Aβ deposition burden in the striatum, as indicated by 6F/3D and other antibodies (especially anti‐Aβ_42_, Aβ_43_, and Aβ_Np3E_ antibodies), was significant in the AD and LBD groups, but comparatively smaller in the PSP group. Additionally, cluster analysis revealed a relatively homogeneous spectrum of deposited Aβ peptides among cases in the PSP group, whereas notable heterogeneity was observed among cases in the AD and LBD groups. These findings suggest a similarity in the progression of Aβ pathology between LBD and AD, while PSP exhibits distinct characteristics. Importantly, the significant presence of highly neurotoxic Aβ variants, such as Aβ_42_, Aβ_43_, and Aβ_Np3E_, in the plaques of LBD patients suggests their potential as promising targets for treatment with anti‐Aβ antibodies. Importantly, the striatum is affected by astrocytic tau pathology in PSP, and thus it is tempting to speculate that the altered microenvironment associated with this contributes to distinct processing of Aβ.

It should be noted that LBD cases tended (*p* = 0.07) to have less cored plaque formation than AD and PSP cases, despite the presence of Aβ deposition similar to that of AD. The reason for this is unknown and is a subject for further study. Ikonomovic et al. have reported that the intensity of [^18^F]flutemetamol and [^11^C]Pittsburgh Compound‐B positron emission tomography signal is influenced by both diffuse plaques and cored plaques, but total fluorescence output from a cored plaque is approximately three times that from a diffuse plaque of the same volume [80]. Thus, it is suggested that amyloid deposition in the striatum may be underestimated in individuals with LBD.

In conclusion, our data imply that the type and strain of concomitant proteinopathy may affect the spectrum of deposited Aβ in non‐AD NDDs. Interactions between abnormal proteins are speculated to be one of the causes of this phenomenon. The female sex and *APOE* ε4 promote Aβ deposition and may accentuate this difference. Aβ deposition is strongly linked to more rapid cognitive deterioration and earlier mortality in individuals with LBD [81, 82]. Thus, we suggest that individuals with LBD, particularly females with one or more *APOE* ε4 alleles, could be considered candidates for treatment with amyloidocentric drugs, should they show efficacy in slowing cognitive decline in patients with AD. By reducing Aβ deposition, the progression of α‐synuclein pathology may also be inhibited by suppressing the synergistic interaction between the two pathological proteins. Additionally, combining tau‐targeting drugs may further augment their therapeutic effectiveness. Furthermore, we found that the spectrum of Aβ peptides deposited in the striatum differs from that in the neocortex. This might also be influenced by the type of the non‐Aβ protein cytopathology. Therefore, a comprehensive analysis of each anatomical site is considered necessary to enhance our understanding of the effects of Aβ, especially for improving diagnostic accuracy in imaging analysis. Establishing therapeutic strategies that integrate these factors may lead to improved treatment outcomes using amyloid‐centric drugs.

## AUTHOR CONTRIBUTIONS

SI conceived and designed the study, conducted the neuropathological observations, and drafted the manuscript and figures; KY performed the cluster analysis and created Figure 6; JL conducted all immunohistochemistry; ER carried out the genetic analysis; AEL provided the clinical information and reviewed the manuscript; GGK performed the histological evaluation, assessed the clinical data, and provided critical review of the manuscript. All authors have read and approved the final version of the manuscript.

## CONFLICT OF INTEREST STATEMENT

Anthony E. Lang has served as an advisor for AbbVie, AFFiRis, Alector, Amylyx, Aprinoia, Biogen, BioAdvance, BlueRock, Biovie, BMS, CoA Therapeutics, Denali, Janssen, Jazz, Lilly, Novartis, Paladin, Pharma 2B, PsychoGenetics, Retrophin, Roche, Sun Pharma, and UCB; received honoraria from Sun Pharma, AbbVie and Sunovion; received grants from Brain Canada, Canadian Institutes of Health Research, Edmond J Safra Philanthropic Foundation, Michael J. Fox Foundation, the Ontario Brain Institute, Parkinson Foundation, Parkinson Canada, and W. Garfield Weston Foundation; is serving as an expert witness in litigation related to paraquat and Parkinson's disease, received publishing royalties from Elsevier, Saunders, Wiley‐Blackwell, Johns Hopkins Press, and Cambridge University Press. Gabor G. Kovacs has served as an advisor for Biogen; received royalty for 5G4 synuclein antibody and publishing royalties from Wiley, Cambridge University Press and Elsevier, received grants from Edmond J Safra Philanthropic Foundation, Rossy Foundation, Michael J. Fox Foundation, Parkinson Canada, Canada, Canada Foundation for Innovation, MSA Coalition, and National Institutes of Health (NIH). The interests listed for Anthony E. Lang and Gabor G. Kovacs have no relevance for the manuscript. Shojiro Ichimata, Koji Yoshida, and Ekaterina Rogaeva do not report any competing interests.

## ETHICS STATEMENT

All brains had been obtained at autopsy through appropriate consenting procedures with Local Ethical Committee approval. This study was approved by the UHN Research Ethics Board (No. 20‐5258) and University of Toronto (No. 39459) and was performed per the ethical standards established in the 1964 Declaration of Helsinki, updated in 2008.

## Supporting information


**Data S1.** Supporting Information.

## Data Availability

All data used in this study are available for review upon request.
