# Supplementary material for: The molecular spectrum of amyloid‐beta (Aβ) in neurodegenerative diseases beyond Alzheimer's disease
Source: Brain Pathol. 2023 Aug 31;34(1):e13210. doi: 10.1111/bpa.13210 (PMC10711260; doi:10.1111/bpa.13210)
Supplement: Supplementary file 1 — Data S1. Supporting Information. [file BPA-34-e13210-s001.pdf]

Online supplementary data

The molecular spectrum of amyloid-beta (Aβ) in neurodegenerative diseases beyond Alzheimer's disease

Shojiro Ichimata,[1,2,3] Koji Yoshida,[1,2,3] Ekaterina Rogaeva,[1] Anthony E. Lang,[1,4], Gabor G. Kovacs[1,2,4,5]

Author affiliations:

[1] Tanz Centre for Research in Neurodegenerative Disease, University of Toronto, Toronto, Ontario, ON M5T 0S8, Canada;

[2] Department of Laboratory Medicine and Pathobiology, University of Toronto, Toronto, Ontario, ON M5S 1A8, Canada;

[3] Department of Legal Medicine, Faculty of Medicine, University of Toyama, Sugitani, Toyama, 930-0194, Japan;

[4] Edmond J Safra Program in Parkinson’s Disease and Rossy Program in Progressive Supranuclear Palsy, Toronto Western Hospital, Toronto, Ontario, ON M5T 2S8, Canada;

[5]Laboratory Medicine Program and Krembil Brain Institute, University Health Network, Toronto, Canada

Supplementary Table S1. All analysis results in the temporal lobe.

Case #	Age	Sex	*APOE*	*MAPT*	ABC score	LPC level	CAA type	CGP[a]	6F/3D (P/V)	6F/3D (Q)	$A\beta_{38}$ (P/V)	$A\beta_{39}$ (P/V)	$A\beta_{40}$ (P/V)	$A\beta_{40}$ (Q)	$A\beta_{42}$ (P/V)	$A\beta_{42}$ (Q)	$A\beta_{43}$ (P/V)	$A\beta_{43}$ (Q)	$A\beta_{Np3E}$ (P/V)	$A\beta_{Np3E}$ (Q)	$A\beta_{pSer8}$ (P/V)	AT8 (SQ)
LBD1	73	M	3/4	1/1	A2B1C2	NC	2	0	2/2	1.9	2/1	1/1	2/2	3.8	3/2	11.1	2/1	2.4	3/2	7.1	2/1	1
LBD2	90	F	3/4	1/1	A3B2C1	LM	1	0	3/3	3.7	1/3	1/3	2/3	1.2	2/2	2.1	3/2	3.6	3/3	6.4	1/2	1
LBD3	78	M	3/4	1/2	A3B2C2	NC	2	0	2/3	NE	2/2	1/2	2/3	NE	3/2	NE	2/1	NE	3/2	NE	2/2	1
LBD4	70	M	3/3	1/2	A3B2C2	NC	2	0	3/1	3.0	2/1	1/0	2/0	1.2	4/0	9.4	3/0	3.7	3/0	3.4	2/1	1
LBD5	59	F	3/4	2/2	A3B3C3	NC	0	0	3/0	4.0	1/0	1/0	3/0	1.8	4/0	11.6	3/0	4.4	3/0	3.6	1/0	3
LBD6	79	F	3/4	1/2	A3B2C2	NC	2	0	3/3	3.2	1/2	1/1	2/3	1.1	4/2	11.1	3/2	3.0	2/2	2.3	1/1	2
LBD7	82	F	3/4	1/1	A2B3C3	NC	2	1	3/3	3.0	2/2	1/2	3/3	2.6	4/3	9.6	3/1	5.8	3/3	3.6	1/2	4
LBD8	76	M	N/A	NA	A2B2C1	NC	1	2	2/3	NE	1/0	1/0	2/3	NE	3/3	NE	2/3	NE	2/3	NE	2/1	1
LBD9	71	M	3/4	1/2	A2B2C3	NC	0	0	2/0	1.4	1/0	1/0	2/0	1.1	4/0	11.5	3/0	3.3	2/0	1.7	2/0	2
LBD10	73	M	3/3	1/1	A2B3C2	NC	2	0	3/3	2.7	1/2	1/2	2/3	1.5	3/2	6.8	3/2	3.8	2/2	2.1	1/2	3
LBD11	79	M	4/4	1/1	A3B3C3	NC	2	4	3/4	5.5	2/3	2/3	3/4	3.4	4/3	13.3	3/3	6.2	3/3	5.1	2/3	4
LBD12	84	M	3/4	1/1	A2B2C2	LM	0	0	2/0	NE	1/0	1/0	2/0	NE	3/0	NE	3/0	NE	3/0	NE	1/1	2
LBD13	78	F	3/4	1/2	A3B3C3	NC	0	0	3/0	2.4	1/0	1/0	3/0	1.9	4/0	10.5	3/0	5.4	3/0	4.0	1/0	3
LBD14	82	F	3/4	1/2	A3B3C3	LM	2	3	3/4	5.6	2/3	1/3	3/3	2.8	4/3	11.7	4/1	9.3	3/3	3.5	1/3	4
LBD15	80	M	3/4	2/2	A2B3C2	LM	1	2	3/4	4.3	1/4	1/4	3/4	NA[b]	3/4	6.3	3/3	4.1	2/4	2.7	1/4	4
LBD16	88	F	4/4	1/2	A2B2C2	LM	1	3	3/4	8.0	2/4	2/4	3/4	4.8	4/4	14.8	4/3	8.6	3/4	8.6	2/3	4
LBD17	73	M	3/4	1/1	A2B2C2	NC	0	0	2/0	0.9	2/0	1/1	2/1	0.9	3/1	8.1	3/0	2.5	2/1	1.4	1/0	2
LBD18	65	F	3/4	1/2	A3B1C2	NC	0	0	2/0	1.7	1/0	1/0	2/0	0.7	4/0	10.6	3/0	3.8	2/0	2.3	2/0	1
LBD19	76	F	4/4	1/1	A3B3C3	NC	2	2	3/3	2.4	1/2	1/2	2/3	1.2	4/3	9.5	3/1	3.0	2/3	2.2	1/2	3
LBD20	92	F	3/4	1/2	A3B3C3	NC	1	2	3/3	4.8	1/2	1/1	3/3	2.0	4/4	15.9	3/1	6.6	3/2	5.7	1/3	3
LBD21	86	F	3/4	1/1	A3B3C3	LM	2	3	3/3	4.0	2/3	1/3	3/3	4.3	4/2	9.1	3/0	5.1	3/2	3.0	2/2	4
LBD22	94	F	3/3	1/2	A2B2C2	NC	1	0	2/1	NE	1/0	1/0	2/0	NE	4/0	NE	4/0	NE	3/1	NE	1/0	2
LBD23	85	M	3/3	1/1	A2B1C2	NC	0	0	2/0	1.6	1/0	1/1	2/0	1.3	3/0	7.9	3/0	4.4	2/0	2.2	1/0	1
LBD24	72	M	3/4	1/1	A3B3C3	NC	2	0	3/1	2.7	1/1	1/1	3/2	2.1	4/1	11.1	3/1	4.2	3/1	2.7	0/0	3
LBD25	85	F	4/4	1/1	A3B3C3	LM	2	3	3/3	5.3	2/3	1/3	3/3	3.2	4/2	17.0	4/2	11.9	4/3	8.6	0/1	4
LBD26	72	M	4/4	1/1	A3B3C3	NC	2	3	3/3	3.5	3/3	2/3	3/3	3.6	4/3	9.3	3/2	5.8	3/3	4.1	1/3	3
LBD27	73	F	3/4	1/2	A2B3C3	NC	2	1	3/3	2.9	2/3	1/3	3/3	2.0	4/3	11.7	4/2	4.8	3/3	3.2	1/3	3
LBD28	76	M	2/4	1/2	A3B1C2	NC	1	0	2/2	1.6	2/2	1/2	3/3	2.3	3/3	9.5	2/3	1.7	2/3	2.4	1/2	1
LBD29	73	F	3/4	1/2	A2B3C2	LM	2	3	3/3	8.1	1/3	1/3	3/3	6.6	4/3	23.1	4/1	16.9	3/3	7.3	1/2	4
LBD30	82	F	3/4	1/2	A2B2C2	NC	0	0	3/0	2.7	1/0	1/0	1/0	0.2	4/0	10.9	3/0	4.1	2/0	1.8	1/0	3
LBD31	62	F	3/3	1/1	A3B3C2	NC	0	0	3/0	3.0	1/1	1/1	3/1	1.8	4/0	9.2	3/0	3.2	3/1	3.0	1/0	3
LBD32	69	F	4/4	1/2	A3B3C2	NC	2	1	3/3	4.2	1/2	1/2	3/3	5.4	4/2	13.5	3/0	6.1	3/2	5.3	1/2	3
LBD33	74	M	NA	NA	A3B2C2	NC	2	1	3/2	NE	1/1	1/1	2/2	NE	4/1	NE	3/0	NE	3/1	NE	1/1	2
LBD34	79	M	3/4	1/1	A2B2C2	NC	0	0	3/0	NE	1/0	0/0	2/0	NE	4/0	NE	3/0	NE	3/0	NE	1/0	1
LBD35	56	M	4/4	1/1	A2B3C2	NC	2	1	3/3	3.0	1/3	1/3	3/3	1.9	4/3	11.8	3/2	4.2	3/3	4.2	2/2	4
LBD36	65	F	3/4	1/2	A3B3C3	LM	2	4	3/3	7.2	2/3	2/3	4/3	11.4	4/3	19.0	4/1	12.0	4/3	10.1	2/3	4
PSP1	NA	M	3/3	1/1	A2B2C2	Neg	0	0	2/0	1.3	1/1	1/1	2/0	0.6	3/0	5.9	2/0	1.4	3/0	3.9	2/0	2
PSP2	78	F	2/4	1/1	A3B3C3	Neg	2	0	2/3	1.1	1/3	1/3	2/4	1.2	4/3	18.0	3/3	1.8	3/3	2.3	2/3	4

PSP3	79	M	2/4	1/2	A3B1C2	Neg	1	0	2/3	NE	0/3	0/3	1/3	NE	3/3	NE	3/2	NE	3/3	NE	1/3	1
PSP4	81	M	3/4	1/1	A2B2C1	BS	1	0	2/2	0.7	1/2	1/2	2/2	0.7	3/2	2.4	2/2	1.3	2/2	1.0	1/2	1
PSP5	84	F	3/4	1/1	A3B1C2	Neg	2	0	2/2	NE	1/2	1/1	2/2	NE	3/2	NE	2/1	NE	3/2	NE	1/1	1
PSP6	79	F	3/4	1/1	A2B1C2	BS	2	0	2/2	NE	2/1	1/1	2/2	NE	3/2	NE	2/2	NE	2/2	NE	2/1	1
PSP7	74	F	3/4	1/1	A2B2C2	Neg	0	0	2/0	1.5	1/0	1/0	2/0	0.4	3/0	8.4	3/0	1.9	3/0	2.9	2/0	2
PSP8	93	F	NA	NA	A2B2C2	LM	2	0	3/1	3.5	1/1	1/0	2/2	0.7	3/1	7.1	3/0	2.0	3/0	3.1	1/1	1
PSP9	82	F	3/4	1/1	A3B2C2	AM	2	0	2/2	1.8	1/2	1/1	2/2	0.8	3/2	8.2	3/0	2.1	3/2	2.4	1/2	3
PSP10	85	M	3/3	1/1	A3B2C3	LM	2	1	3/2	2.6	1/1	1/2	2/2	0.5	3/2	6.6	3/1	5.2	3/2	4.1	1/1	3

Abbreviations: AM, amygdala-predominant; *APOE*, apolipoprotein E genotype; BS, brainstem-predominant; CAA, cerebral amyloid angiopathy; CGP, coarse-grained plaque grade (0 [none] to 4 [severe]); F, female; LBD, Lewy body disease; LM, limbic; LPC, Lewy pathology consensus criteria; NA, not available; M, male; NC, neocortical; NE, not evaluated; Neg, negative; P, plaque pathology grade (0 [none] to 4 [severe]); PSP, progressive supranuclear palsy; Q, quantitative value; SQ, semiquantitative analysis (0 [none] to 4 [severe]); V, vascular pathology (CAA) grade (0 [none] to 4 [severe])

[a] Presence or absence of the CGP in the temporal lobe was evaluated using specimens immunostained for Aβ (6F/3D).

[b] Quantitative analysis was not feasible due to the intense background immunoreactivity.

Supplementary Table S2. Summary of clinical information and all Aβ analysis results in the striatum.

						6F/3D				$A\beta_{38}$		$A\beta_{39}$		$A\beta_{40}$				$A\beta_{42}$				$A\beta_{43}$				$A\beta_{Np3E}$				$A\beta_{pSer8}$	
Case #	Age	Sex	*APOE*	ABC score	CP	C-SQ	C-Q[a]	P-SQ	P-Q[a]	C-SQ	P-SQ	C-SQ	P-SQ	C-SQ	C-Q[a]	P-SQ	P-Q[a]	C-SQ	C-Q[a]	P-SQ	P-Q[a]	C-SQ	C-Q[a]	P-SQ	P-Q[a]	C-SQ	C-Q[a]	P-SQ	P-Q[a]	C-SQ	P-SQ
LBD1	73	M	3/4	A2B1C2	1	3	3.2	4	7.6	0	0	1	0	2	0.9	2	0.7	3	5.2	3	6.1	3	2.4	3	3.1	3	6.1	3	7.4	1	1
LBD2	90	F	3/4	A3B2C1	0	3	3.5	3	5.2	0	0	0	0	1	0.5	1	0.2	4	9.3	4	12.8	3	6.5	4	7.8	4	14.8	4	18.3	2	1
LBD3	78	M	3/4	A3B2C2	0	3	2.0	3	2.4	0	0	0	0	1	0.2	1	0.2	3	4.3	3	5.3	3	2.3	3	2.7	3	6.5	3	8.3	2	2
LBD4	70	M	3/3	A3B2C2	1	4	9.7	4	12.5	0	0	0	0	3	2.6	3	3.3	4	12.2	4	15.4	3	5.1	3	6.6	4	13.0	4	15.7	3	3
LBD5	59	F	3/4	A3B3C3	1	4	10.4	4	12.6	0	0	0	0	3	1.5	2	1.4	4	14.6	4	17.2	4	8.3	4	7.8	4	11.6	4	11.2	2	2
LBD6	82	F	3/4	A2B3C3	1	3	5.1	4	7.6	0	0	0	0	1	0.1	1	0.6	4	9.6	4	13.1	3	4.4	3	5.2	4	7.0	4	9.8	2	2
LBD7	76	M	NA	A2B2C1	2	3	1.8	3	2.5	0	0	0	0	1	0.3	1	0.5	3	1.6	3	2.5	2	0.9	2	1.2	2	1.3	2	1.4	1	1
LBD8	82	F	3/4	A3B3C3	1	3	6.5	4	7.0	0	0	0	0	3	1.9	3	2.1	4	10.1	4	11.8	3	4.8	4	7.3	4	8.5	4	8.8	1	1
LBD9	76	F	4/4	A3B3C3	0	4	13.5	4	16.5	0	0	0	0	2	1.0	2	0.8	4	10.5	4	13.9	3	5.0	3	5.9	4	11.5	4	11.8	1	1
LBD10	85	M	3/3	A2B1C2	1	3	2.9	3	3.3	0	0	0	0	1	0.4	1	0.2	3	5.5	3	5.3	3	5.1	3	4.4	3	5.3	3	4.4	1	1
LBD11	76	M	2/4	A3B1C2	0	3	4.1	3	5.5	0	0	0	0	1	0.6	2	1.0	3	4.2	4	6.6	3	2.1	3	2.6	3	2.2	3	2.9	2	1
LBD12	73	F	3/4	A2B3C2	1	4	10.5	4	11.6	0	0	0	0	2	1.3	1	0.2	4	11.7	4	14.7	4	8.6	4	12.1	3	5.7	3	8.2	1	1
LBD13	82	F	3/4	A2B2C2	0	3	3.2	3	3.6	0	1	1	0	1	0.5	2	1.1	3	5.5	4	7.7	3	3.9	3	5.4	3	5.6	3	5.3	1	1
LBD14	62	F	3/3	A3B3C2	1	4	13.3	4	17.5	0	0	0	0	3	1.8	3	2.2	4	10.0	4	14.5	3	5.2	3	6.4	4	9.7	4	11.9	2	2
LBD15	69	F	4/4	A3B3C2	1	3	3.8	3	4.5	0	0	0	0	2	0.8	2	1.0	4	8.6	4	12.4	3	5.1	3	5.7	4	10.6	4	12.3	1	1
AD1	66	F	NA	A3B3C3	2	3	2.7	3	2.0	1	1	0	0	2	0.9	2	0.5	4	7.6	4	7.6	3	3.8	3	4.3	4	8.1	4	8.1	3	3
AD2	82	M	NA	A3B3C3	0	3	4.0	3	6.7	0	0	0	0	1	0.5	1	1.0	4	8.7	4	15.4	3	4.6	4	8.3	4	14.0	4	22.0	2	2
AD3	82	F	NA	A3B3C3	3	3	6.3	4	8.1	0	0	0	0	2	1.2	2	1.1	4	7.1	4	8.3	3	4.4	3	4.8	3	5.9	3	7.0	3	3

AD4	79	F	NA	A3B3C3	1	4	13.4	4	19.2	0	0	0	0	3	2.9	3	2.4	4	16.3	4	20.3	4	7.3	4	8.4	4	11.7	4	15.6	2	2
AD5	78	M	NA	A3B3C3	2	3	5.3	4	6.4	0	0	0	0	3	2.2	3	3.4	4	15.2	4	22.0	3	5.7	4	8.8	4	12.5	4	18.8	2	2
AD6	76	F	NA	A3B3C3	1	3	3.8	3	5.4	0	0	0	0	2	1.3	2	0.9	4	10.9	4	15.0	3	6.2	3	6.2	4	9.3	4	11.1	1	1
PSP1	NA	M	3/3	A2B2C2	2	3	5.5	3	6.2	0	0	0	0	2	1.1	1	0.4	3	6.5	4	8.3	3	3.3	3	3.2	4	8.9	4	9.4	2	2
PSP2	78	F	2/4	A3B3C3	1	4	8.4	4	9.4	1	0	0	0	2	1.3	2	1.4	4	9.6	4	12.3	3	5.4	3	6.3	4	9.6	4	11.6	3	3
PSP3	81	M	3/4	A2B2C1	1	3	1.5	3	2.4	0	0	0	0	2	1.2	2	1.1	3	3.6	3	6.5	3	2.1	3	2.7	3	3.4	3	3.1	2	2
PSP4	74	F	3/4	A2B2C2	2	3	2.2	3	2.7	0	0	0	0	2	0.5	1	0.4	3	6.5	4	8.7	3	3.0	3	3.6	3	5.7	3	6.2	3	2
PSP5	82	F	3/4	A3B2C2	1	4	8.3	4	9.4	0	0	0	0	2	0.9	2	0.7	3	6.0	4	7.2	3	2.6	3	2.5	3	5.1	3	5.4	2	2

Abbreviations: C, caudate nucleus; CP; cored plaque grade (0 [none] to 3 [frequent]); Int, intermediate; LM, limbic; P, putamen; SQ, semiquantitative analysis (0 [none] to 4 [severe])

[a] Mean value (10 locations).

Supplementary Table S3. Summary of the clinical, pathological, and quantitative (Q) results of striatal Aβ analysis in individuals with A score 3.

		LBD	AD	PSP	*P* value[a] All (LBD–AD/LBD–PSP/PSP–AD)
# of cases		9	6	2	
Mean age (range)		73.6 ± 9.1 (59–90)	77.2 ± 5.4 (66–82)	80.0 ± 2.0 (78–82)	0.37
Sex (F/M)		6/3	4/2	2/0	
CP grade (0/A/B/C)		4/5/0/0	1/2/2/1	0/2/0/0	0.12
APOE score (0/1/2)		2/5/2	NA	0/2/0	
6F/3D	Q-CN	7.4 ± 4.4 (1.5–18.1)	5.9 ± 3.7 (1.4–18.3)	8.4 ± 2.0 (5.6–13.8)	**< 0.01** (0.12 /0.18/**< 0.01**)
	Q-Put	9.3 ± 5.5 (1.4–22.6)	8.0 ± 5.6 (1.3–22.5)	9.4 ± 1.6 (6.4–12.2)	**0.04** (0.38/0.36/**0.04**)
$A\beta_{40}$	Q-CN	1.2 ± 0.9 (0.0–3.9)	1.5 ± 1.1 (0.1–4.2)	1.1 ± 0.6 (0.0–2.4)	0.21
	Q-Put	1.4 ± 1.2 (0.0–4.7)	1.6 ± 1.5 (0.1–5.9)	1.1 ± 1.0 (0.1–4.6)	0.42
$A\beta_{42}$	Q-CN	9.3 ± 3.7 (2.8–18.1)	11.0 ± 4.0 (5.3–19.4)	7.8 ± 2.5 (4.0–11.9)	**< 0.01** (0.16/0.21/**< 0.01**)
	Q-Put	12.2 ± 4.2 (5.4–21.4)	14.8 ± 5.8 (5.4–26.9)	9.8 ± 3.0 (5.5–15.2)	**< 0.01** (**0.02**/0.06/**< 0.01**)
$A\beta_{43}$	Q-CN	4.9 ± 2.0 (1.3–9.9)	5.3 ± 1.4 (3.0–9.1)	4.0 ±1.6 (1.4–6.8)	**0.03** (0.78/0.14/**0.03**)
	Q-Put	5.9 ± 2.1 (1.6–10.6)	6.8 ± 2.2 (3.2–12.2)	4.4 ± 2.1 (1.6–8.4)	**< 0.01** (0.14/**0.03**/**< 0.01**)
$A\beta_{Np3E}$	Q-CN	9.8 ± 3.9 (1.5–18.0)	10.2 ± 3.1 (4.4–17.5)	7.3 ± 2.8 (3.9–14.7)	**< 0.01** (1.00/ **< 0.01**/**< 0.01**)
	Q-Put	11.3 ± 4.5 (2.1–21.6)	13.8 ± 6.2 (3.5–29.1)	8.5 ± 3.3 (4.4–13.5)	**< 0.01** (0.18/0.06/**< 0.01**)

Abbreviations: CN, caudate nucleus; Put, putamen

Boldface signifies values that are significant at $P < 0.05$

Supplementary Table S4. Summary of the clinical, pathological, and quantitative (Q) results of striatal Aβ analysis in individuals with A score 2.

		LBD	PSP	*P* value[a]
# of cases		6	3	
Mean age (range)		78.5 ± 4.7 (73–85)	77.5 ± 3.5[b] (74–81)	0.86
Sex (F/M)		3/3	1/2	1.00
CP grade (0/A/B/C)		1/4/1/0	0/1/2/0	0.26
APOE score (0/1/2)		1/4/0[c]	1/2/0	0.79
6F/3D	Q-CN	4.5 ± 3.3 (0.9–17.1)	3.1 ± 1.8 (0.9–6.9)	**0.02**
	Q-Put	6.0 ± 3.3 (1.6–13.1)	3.7 ± 1.8 (1.1–7.1)	**< 0.01**
$A\beta_{40}$	Q-CN	0.6 ± 1.0 (0.0–6.8)	0.9 ± 0.5 (0.1–2.0)	**< 0.01**
	Q-Put	0.6 ± 0.6 (0.0–3.1)	0.6 ± 0.5 (0.0–1.8)	0.48
$A\beta_{42}$	Q-CN	6.5 ± 3.7 (0.9–17.7)	5.5 ± 2.0 (2.2–11.0)	0.29
	Q-Put	8.2 ± 4.6 (1.0–19.1)	7.8 ± 1.8 (4.5–11.3)	0.64
$A\beta_{43}$	Q-CN	4.2 ± 2.6 (0.3–12.3)	2.8 ±0.7 (1.5–4.2)	**< 0.01**
	Q-Put	5.2 ± 3.5 (0.5–14.0)	3.1 ± 0.8 (1.8–4.7)	**< 0.01**
$A\beta_{Np3E}$	Q-CN	5.1 ± 2.1 (0.6–8.5)	6.0 ± 2.4 (2.7–11.1)	0.31
	Q-Put	6.1 ± 3.1 (0.7–13.4)	6.3 ± 2.9 (1.8–11.2)	0.78

Abbreviations: CN, caudate nucleus; Put, putamen

Boldface signifies values that are significant at *P* < 0.05

[a] LBD cases vs. PSP cases (Fisher's exact test or Mann-Whitney U test)

[b] Not available in one case

[c] Not available in one case

Supplementary Table S5. Summary of the clinical and pathological findings of each group in the temporal lobe

		Low Aβ group	High Aβ group	P value[a]
Total # of cases		28	8	
# of LBD//PSP cases (%)		21/7 (78/100)	8/0 (100/0)	
Sex (F/M)		15/13	7/1	0.12
Mean age (range)		75.8 ± 5.2 (66–85)[b]	70.1 ± 7.8 (59–79)	0.16
	ε2	2 (4)	0	
APOE allele frequency, all (%)	ε3	28 (52)	4 (25)	**0.046**
	ε4	24 (44)	12 (75)	
	ε2	1	0	
APOE allele frequency, LBD (%)	ε3	21	4 (25)	0.08
	ε4	20	12 (75)	
NIA-AA level	LBD	4/10/7	0/2/6	**0.04**
(Low/Int/High)	PSP	0/6/1	0	
Mean 6F/3D burden (range)		2.4 ± 0.9 (0.7–4.0)	6.1 ± 1.4 (4.2–8.1)	**< 0.01**
Mean Aβ40 burden (range)		1.6 ± 1.0 (0.2–4.3)	5.0 ± 2.8 (2.0–11.4)	**< 0.01**
Mean Aβ42 burden (range)		9.3 ± 3.0 (2.1–18.0)	16.0 ± 3.4 (11.7–23.1)	**< 0.01**
Mean Aβ43 burden (range)		3.5 ± 1.3 (1.3–5.8)	9.7 ± 3.5 (6.1–16.9)	**< 0.01**
Mean AβNp3E burden (range)		3.1 ± 1.3 (1.0–7.1)	6.8 ± 2.1 (3.5–10.1)	**< 0.01**

Boldface signifies values that are significant at $P < 0.05$

[a] Aβ-low group vs. Aβ-high group (Fisher's exact test or Mann-Whitney U test)

[b] Not available in one PSP case.

Supplementary Table S6. Summary of the clinical and pathological findings of each group in the striatum

		Low Aβ group	Int Aβ group	High Aβ group	P value[a] All (Low-Int/Low-High/Int-High)
Total # of cases		12	8	6	
Sex (F/M)		5/7	7/1	4/2	
Mean age (range)		77.7 ± 5.2 (66–85)[b]	79.0 ± 6.1 (69–90)	70.1 ± 7.8 (59–79)	0.157
# of cases in each group (LBD/AD/PSP; %)		6/2/4 (40/33/80)	5/2/1 (33/33/20)	4/2/0 (27/33/0)	
NIA-AA level (Low/Int/High)	LBD	3/2/0	0/3/2	0/1/3	
	AD	0/0/2	0/0/2	0/0/2	
	PSP	0/4/0	0/0/1	0	
Mean 6F/3D burden (range)	CN	3.7 ± 2.2 (0.9–13.8)	5.7 ± 2.9 (1.4–17.1)	10.9 ± 3.5 (3.9–18.3)	**< 0.01** (**< 0.01**/**< 0.01**/**< 0.01**)
	Put	4.6 ± 2.7 (1.1–12.2)	7.2 ± 2.4 (3.7–13.1)	14.1 ± 4.8 (4.1–22.6)	**< 0.01** (**< 0.01**/**< 0.01**/**< 0.01**)
Mean Aβ40 burden (range)	CN	0.7 ± 0.5 (0.0–2.0)	1.0 ± 1.2 (0.0–6.8)	2.0 ± 0.8 (0.1–3.9)	**< 0.01** (1.00/**< 0.01**/**< 0.01**)
	Put	0.7 ± 0.5 (0.0–3.1)	0.9 ± 1.2 (0.0–4.7)	2.3 ± 1.3 (0.1–5.9)	**< 0.01** (1.00/**< 0.01**/**< 0.01**)
Mean Aβ42 burden (range)	CN	5.3 ± 2.0 (0.9–11.0)	9.8 ± 2.3 (6.0–17.7)	13.1 ± 3.2 (6.9–19.4)	**< 0.01** (**< 0.01**/**< 0.01**/**< 0.01**)
	Put	6.7 ± 2.1 (1.0–11.3)	13.4 ± 2.2 (9.0–19.3)	17.2 ± 3.9 (9.9–26.9)	**< 0.01** (**< 0.01**/**< 0.01**/**< 0.01**)
Mean Aβ43 burden (range)	CN	3.0 ± 1.3 (0.3–6.4)	5.7 ± 1.8 (2.8–12.3)	6.1 ± 1.5 (3.4–9.9)	**< 0.01** (**< 0.01**/**< 0.01**/0.49)
	Put	3.4 ± 1.3 (0.5–6.7)	7.4 ± 2.4 (3.7–14.0)	7.3 ± 1.6 (4.8–12.2)	**< 0.01** (**< 0.01**/**< 0.01**/1.00)
Mean AβNp3E burden (range)	CN	5.3 ± 2.3 (0.6–11.1)	9.9 ± 3.4 (3.1–18.0)	11.7 ± 2.1 (5.8–16.0)	**< 0.01** (**< 0.01**/**< 0.01**/**< 0.01**)
	Put	5.7 ± 2.7 (0.7–11.7)	12.8 ± 5.1 (6.6–29.1)	14.2 ± 3.6 (6.7–25.2)	**< 0.01** (**< 0.01**/**< 0.01**/0.08)

Boldface signifies values that are significant at $P < 0.05$

[a] Kruskal-Wallis test with post hoc Bonferroni correction

[b] Not available in one PSP case

Supplementary Figure 1. Histopathological Aβ and tau deposition grading (G) in the temporal lobe.

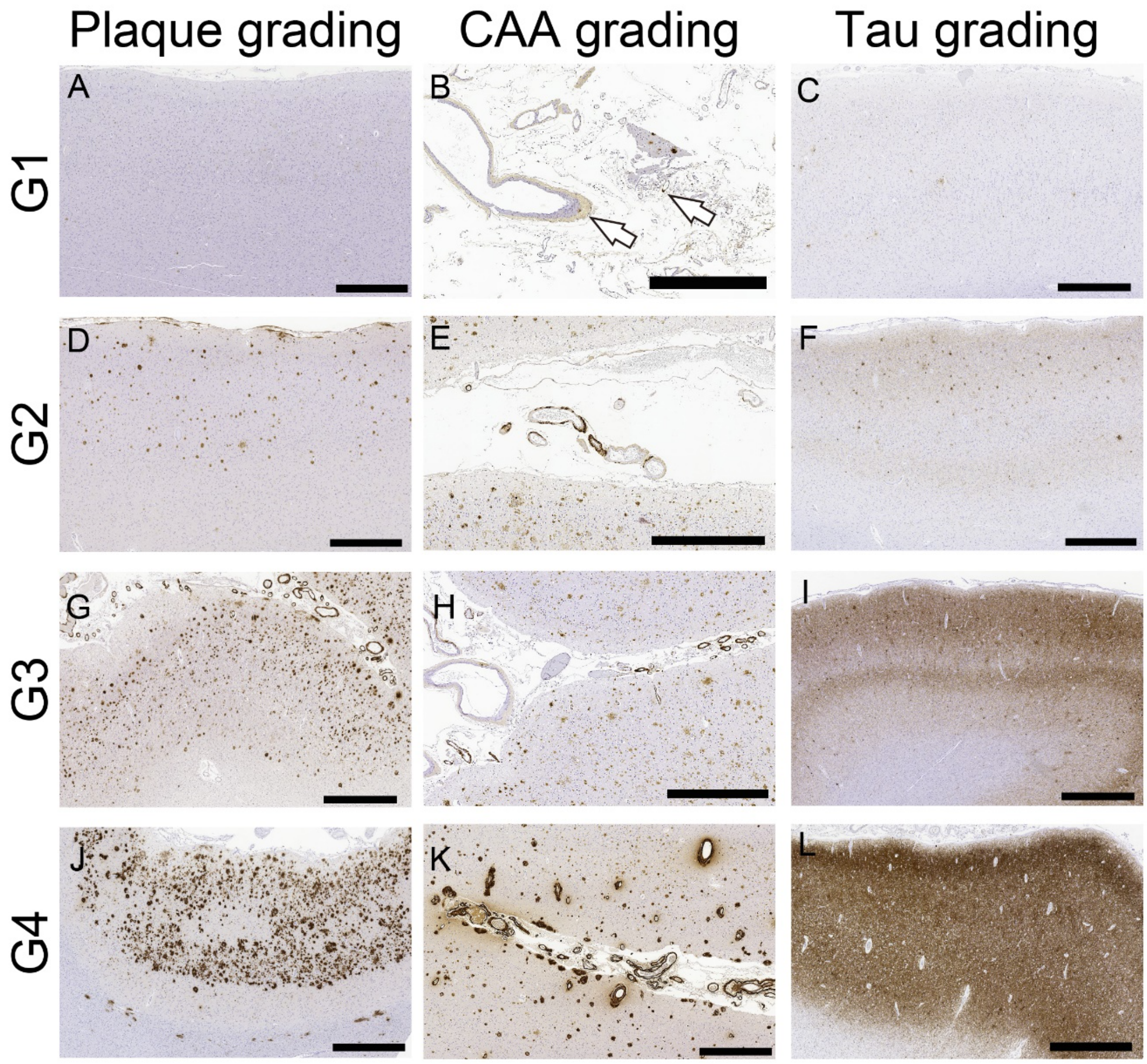

Immunohistochemistry for Aβ (6F/3D) (B, D, E, G, H, K); $A\beta_{40}$ (A); $A\beta_{42}$ (J); phosphorylated tau (AT8) (C, F, I, L). Arrows in panel B indicate small amyloid deposits in the leptomeningeal vessels.

Scale bar = 100 μm (A–L)

Supplementary Figure 2. Representative microphotographs of $A\beta_{pSer8}$ deposition in the striatum.

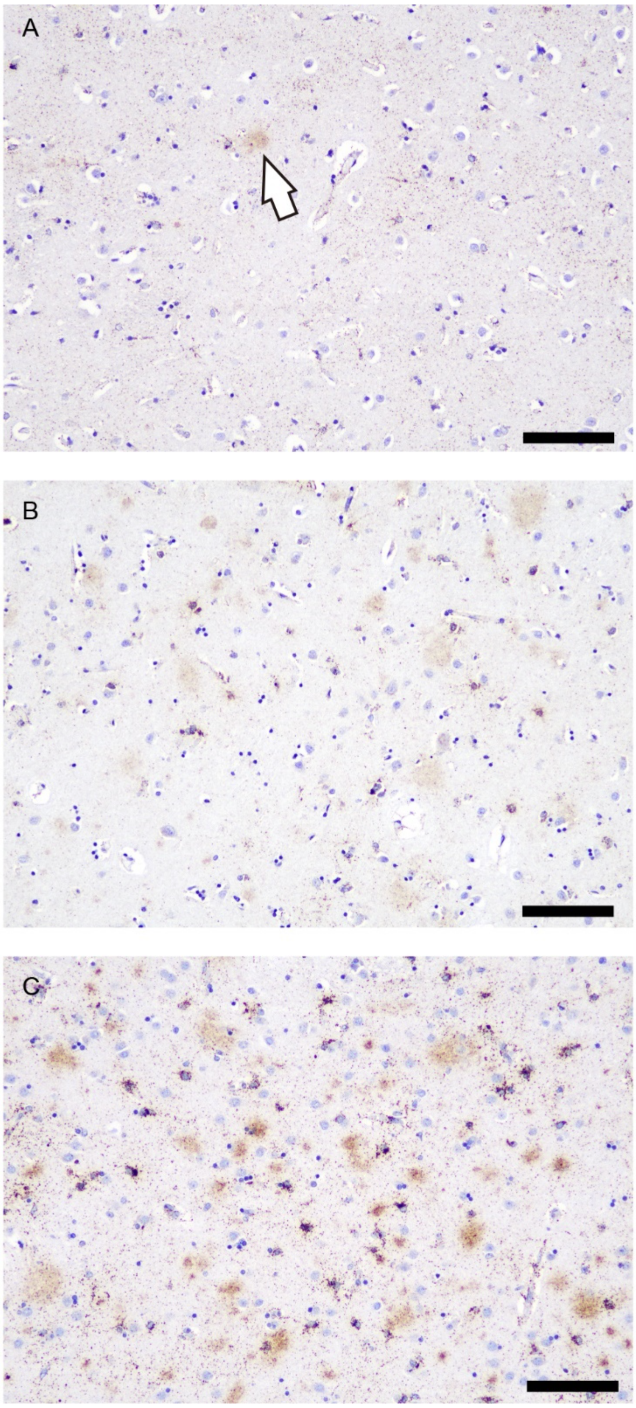

(A) LBD7; (B) LBD7; (C) LBD4. (A) Striatal Aβ deposition grade 1 (arrow indicates the plaque). (B) Striatal Aβ deposition grade 2. (C) Striatal Aβ deposition grade 3. Most of the plaques showed diffuse-type morphology. Note that there are some synaptic-like and astrocytic AβpSer8-immunoreactivity in the background.

Scale bar = 100 μm (A–C)

Supplementary Figure S3. Results of the quantitative Aβ deposition analysis in the striatum in individuals with A score 3.

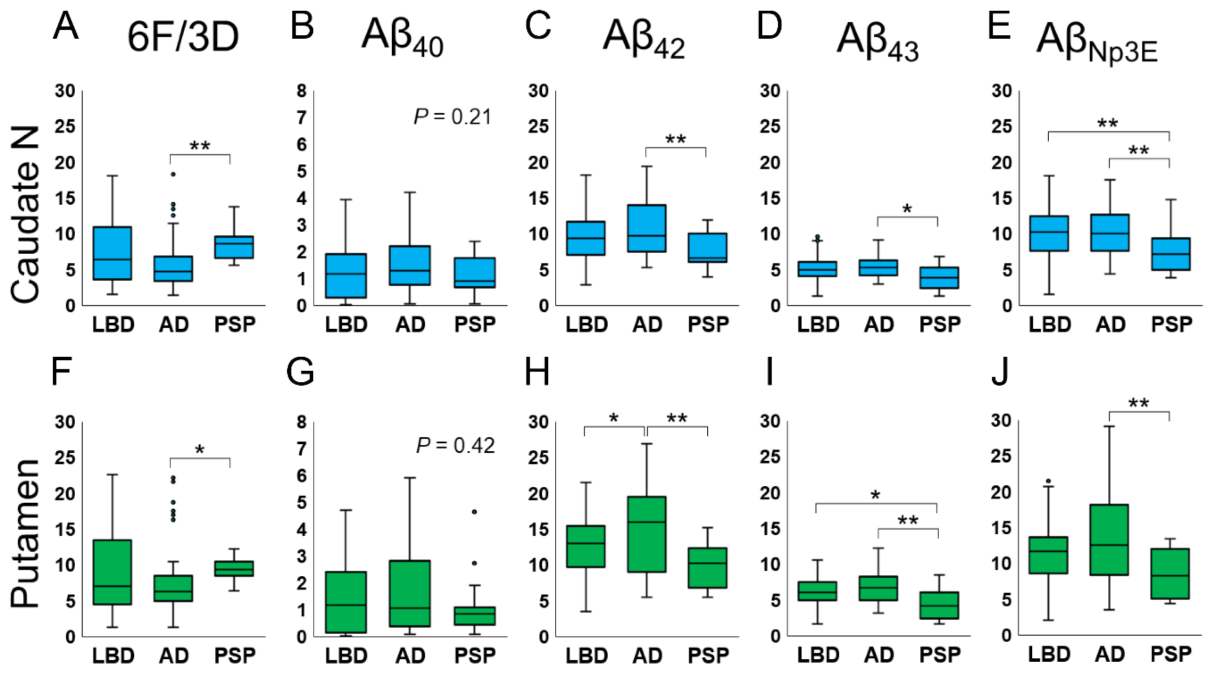

Results of the quantitative analysis of specimens immunostained for 6F/3D (A, F); $A\beta_{40}$ (B, G); $A\beta_{42}$ (C, H); $A\beta_{43}$ (D, I); $A\beta_{Np3E}$ (E, J). * $P < 0.05$; ** $P < 0.01$ (Kruskal-Wallis test with post hoc Bonferroni correction)

Supplementary Figure S4. Results of the quantitative Aβ deposition analysis in the striatum in individuals with A score 2.

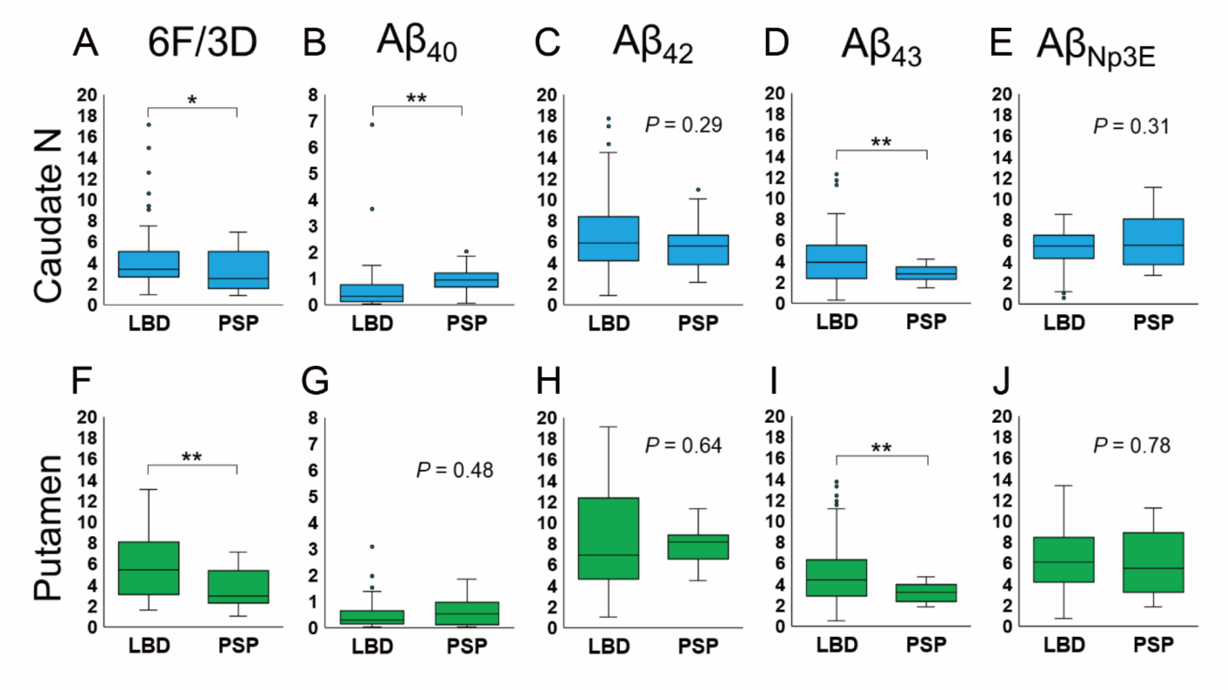

Results of the quantitative analysis of specimens immunostained for 6F/3D (A, F); $A\beta_{40}$ (B, G); $A\beta_{42}$ (C, H); $A\beta_{43}$ (D, I); $A\beta_{Np3E}$ (E, J). * $P < 0.05$; ** $P < 0.01$ (Mann-Whitney U test)
